# Vaccine-expanded plasmablast-like B cells are associated with response to dendritic cell therapy in metastatic melanoma

**DOI:** 10.1186/s13046-026-03731-5

**Published:** 2026-05-23

**Authors:** Marcella Tazzari, Silvia Carloni, Jenny Bulgarelli, Sara Pignatta, Martine Bocchini, Claudia Piccinini, Davide Angeli, Michela Tebaldi, Irene Azzali, Maria Maddalena Tumedei, Filippo Piccinini, Francesca Tauceri, Francesco Limarzi, Fabio Nicolini, Maria Teresa Bochicchio, Milena Urbini, Giovanni Foschi, Nicola Romanini, Francesco de Rosa, Anna Maria Granato, Elena Pancisi, Massimiliano Petrini, Laura Ridolfi

**Affiliations:** 1 Advanced Cellular Therapies and Rare Tumors Unit, IRCCS Istituto Romagnolo Per Lo Studio Dei Tumori (IRST) “Dino Amadori”, Meldola, FC 47014 Italy; 2 Immuno-Gene Therapy Factory, IRCCS Istituto Romagnolo Per Lo Studio Dei Tumori (IRST) “Dino Amadori”, Meldola, FC 47014 Italy; 3 Unit of Biostatistics and Clinical Trials, IRCCS Istituto Romagnolo Per Lo Studio Dei Tumori (IRST) “Dino Amadori”, Meldola, FC 47014 Italy; 4 Department of Medical and Surgical Sciences (DIMEC), University of Bologna, Via G.Massarenti 9, Bologna, 40138 Italy; 5 Scientific Directorate, Unit of Biostatistics and Clinical Trials, IRCCS Istituto Romagnolo Per Lo Studio Dei Tumori (IRST) “Dino Amadori”, Meldola, FC 47014 Italy; 6 General and Oncological Surgery, Morgagni-Pierantoni Hospital, 47121 Forlì, Italy; 7 Pathology Unit, Morgagni-Pierantoni Hospital, AUSL Romagna, Forlì, 47121 Italy; 8 Biosciences Laboratory, IRCCS Istituto Romagnolo Per Lo Studio Dei Tumori (IRST) “Dino Amadori”, 47014 Meldola, Italy; 9 Unit of Medical Genetics, The Greater Romagna Area Hub Laboratory, Piazza Della Liberazione 60, Cesena, 47522 Italy

**Keywords:** Dendritic cell vaccine, Melanoma, B cells, Single cell transcriptomics, Tertiary lymphoid structures

## Abstract

**Background:**

Dendritic Cell Vaccines (DCVax) can induce tumor-specific immune responses, yet their clinical activity remains limited and poorly understood. We sought to identify cellular and molecular features within the vaccine product that are associated with clinical response to monocyte-derived DC vaccines in metastatic melanoma.

**Methods:**

We performed a multi-omics analysis integrating multiparametric flow cytometry, single-cell RNA sequencing of DCVax products, transcriptomic profiling of CD14⁺ monocytes from apheresis, and in situ characterization of pre-treatment melanoma biopsies. Patients were stratified into Responders (Rs) or Non-Responders (NRs) based on best overall response and Delayed-Type Hypersensitivity (DTH) status.

**Results:**

An unanticipated population of CD19⁺ plasmablast-like B cells was identified within the final DCVax products. These B cells, phenotypically distinct from their circulating precursors, were significantly enriched in Rs and mirrored a B-cell-inflamed baseline state characterized by mature Tertiary Lymphoid Structures (mTLS) in pre-treatment tumor lesions. While mature LAMP3⁺ DCs appeared at comparable frequencies across outcomes, LAMP3⁺ DCs from Rs selectively upregulated *HSPA1A/B*, consistent with enhanced antigen-processing programs. Transcriptomic signatures of antibody production in vaccine-resident B cells, together with Fc receptor expression on DCs, support a model in which B-cell activity may contribute to antigen loading and DC functional tuning during vaccine manufacturing, a hypothesis that warrants functional validation.

**Conclusions:**

Our findings reveal a previously unrecognized B-cell component of DCVax biology, suggesting that cooperative DC-B-cell interactions, combined with baseline B-cell/mTLS features, may contribute to shaping vaccine immunogenicity. While causality cannot be established from the present data, these insights offer actionable avenues for enhancing both vaccine manufacturing and patient selection, extending beyond melanoma.

**Supplementary Information:**

The online version contains supplementary material available at 10.1186/s13046-026-03731-5.

## Background

Dendritic cells (DCs) are professional antigen-presenting cells and key orchestrators of the adaptive immune response. Their unique capacity to initiate and shape T cell responses has driven their clinical development as Advanced Therapy Medicinal Products (ATMPs) in cancer immunotherapy, particularly in the form of DC-based vaccines. Despite the heterogeneity of DC manufacturing approaches, most clinically applied products, including sipuleucel-T, the only FDA-approved DC-based vaccine, rely on the in vitro differentiation of circulating monocytes into DC surrogates (monocyte-derived DCs, moDCs) rather than naturally occurring DCs [1]. While their safety profile is well established, durable clinical efficacy has remained limited. DC vaccinations are designed to elicit robust and long-lived anti-tumor T cell responses. Systemic T cell responses are often detectable in vaccinated patients, highlighting the immunogenicity of the approach; however, only a small percentage of patients achieved objective clinical responses [2–4]. Another immunological correlate of vaccine-induced responsiveness, described by our group and others, is the Delayed-Type Hypersensitivity (DTH) reaction, which is consistently positive in patients showing clinical benefit and only occasionally observed in those with progressive disease [3, 5]. To improve the clinical efficacy of DC-based therapies, numerous strategies have been proposed, including enhanced maturation cocktails, antigen-loading techniques (predefined neoantigens vs. agnostic whole tumor lysates), and modulation of metabolic pathways to increase immunostimulatory potential [6–8]. Melanoma has always worked as a prototypical model for immunotherapy, where antigen-loaded DC vaccines have been shown to enhance epitope spreading [9] and favor intratumoral T cell infiltration [4]. Recently, the presence and functional status of DCs have been shown to play a pivotal role in determining the efficacy of Immune Checkpoint Inhibitors (ICIs) [10, 11]. In parallel, tumor-associated B cells, particularly those localized within Tertiary Lymphoid Structures (TLS), have emerged as promising predictive biomarkers of ICI response [12–15]. Within these TLS, the enrichment of mature DCs expressing Lysosomal-Associated Membrane Protein 3 (LAMP3) has also attracted considerable attention due to its association with favorable immunological and clinical outcomes [16–18]. However, while single-cell transcriptomic approaches have provided valuable insights into systemic and intratumoral DC heterogeneity [1, 19, 20], they have not been applied to dissect the cellular complexity of ex vivo manufactured moDC vaccines. In this study, we combine Single-cell RNA Sequencing (scRNA-seq) and flow cytometry to analyze both the final moDC vaccine product and the basal immunity from patients with advanced melanoma treated with moDC-based vaccines. Our study focused on dissecting the cellular and molecular composition of Good Manufacturing Practice (GMP)-manufactured DC vaccines and their matched peripheral blood samples in relation to clinical response, aiming to uncover factors potentially linked to improved outcomes. In doing so, we identified a previously unknown plasmablast-like B-cell population within the DC vaccine product, suggesting an additional layer of complexity in the mechanisms underlying vaccine efficacy.

## Methods

### Patients and clinical outcomes

A total of 21 patients were treated in a compassionate use program with dendritic cell (DC) vaccination at Morgagni Hospital (Forlì, FC, Italy) and IRCCS-IRST (Meldola, FC, Italy) between 2002 and 2013 [3]. Patient characteristics are reported in Table 1. All patients were given intradermally mature autologous DC pulsed with autologous tumor lysate (ATL) or autologous tumor homogenate (ATH) and keyhole limpet hemocyanin (KLH). The median age was 51 years (range 27–78), with a balanced gender distribution (11 males and 10 females). Antitumor immune response to the Dendritic Cell Vaccines (DCVax) was evaluated in vivo with the DTH test. Briefly, serial concentrations (100, 50, 20, 10, and 5 µg) of ATL/ATH and KLH were intradermally injected into the forearms of patients, and erythema and induration were recorded after at least 24 h. DTH was considered positive if the area of erythema and/or induration measured at least 5 mm at any antigen concentration. Twelve patients exhibited an immunoresponsive profile to the vaccine, as determined by a positive DTH test following at least four induction immunizations. The remaining nine patients were DTH-negative. According to Response Evaluation Criteria in Solid Tumors (RECIST) 1.1 criteria, BOR included: complete response (CR) in one patient, partial response (PR) in three, stable disease (SD) in four, and progressive disease (PD) in thirteen patients. The median duration of response was 13.5 months (range 4—156), and median overall survival (OS) was 13 months (range 3—159) with 2 Rs and 1 NR patients still alive. Six patients received the DCVax as first-line therapy, while eight had been previously treated with at least one line of chemotherapy and/or cytokine-based therapies, including biochemotherapy (BioCT) or low-dose interferon-alpha (ldIFN). All patients had at least one metastatic site, involving lymph nodes, soft tissue, lung, or visceral organs. Seven patients had evidence of metastases in two distinct anatomical sites. The study was conducted in accordance with the Declaration of Helsinki. A summary of sample availability for the different technologies used is shown in Supplementary Table 1.

**Table 1:**
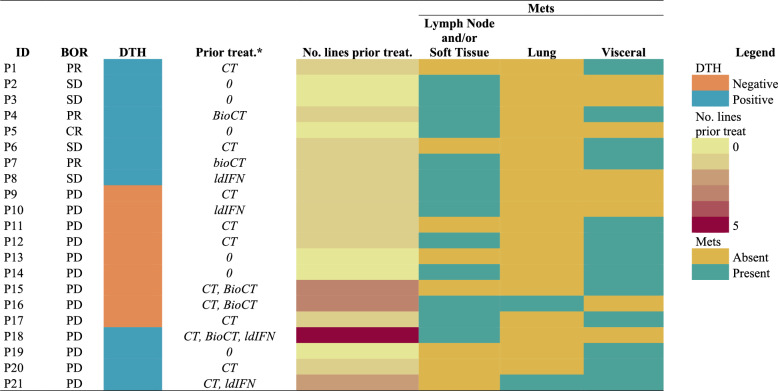
Patient characteristics

*BOR* Best Overall Response, *CR* Complete Response, *PR* Partial Response, *SD* Stable Disease, *PD* Progressive Disease, *DTH* Delayed-Type Hypersensitivity, *Prior treat* Prior systemic treatment, *CT* Chemotherapy, *BioCT* Biochemotherapy, *IdIFN* Low-dose Interferon

^*^in order of administration; No. lines prior treat, Number of lines of prior systemic treatments; Mets, Metastases

### Generation and administration of DCs

**Fig. 1:**
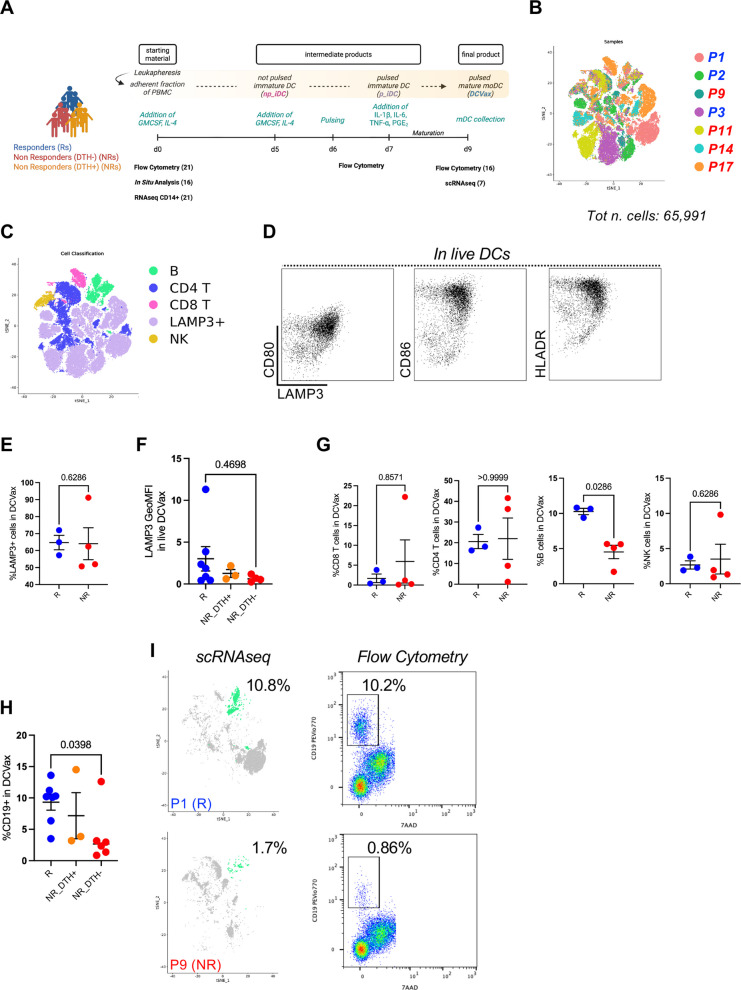
Transcriptomic and Phenotypic profiling of DCVax in relation to clinical outcome. **A** Schematic representation of the ex vivo monocyte-to-DC manufacturing workflow, summarizing the analyzed cell stages and the corresponding analytical methods used in this study. Patients were grouped as Responders (Rs) or Non-Responders (NRs; NR_DTH⁺ and NR_DTH⁻). Created with BioRender. **B** t-distributed stochastic neighbor embedding (*t*-SNE) plot of 65,991 single cells from seven representative patients following quality control and filtering, colored by patient ID. **C** *t*-SNE plot displaying cell cluster classification across all samples. Clusters were identified using the *Seurat* R package and annotated with *clustifyr*. **D** Representative flow cytometry plots showing expression of CD86, CD80, and HLA-DR in live DCs, stratified by LAMP3 expression to identify mature DCs. **E** Scatter plots comparing the frequency of selected intra-vaccine LAMP3⁺ DCs**. F** Scatter plot of the geometric mean fluorescence intensity (GeoMFI) of LAMP3 in DC products from Rs (*n* = 7, blue), NR_DTH⁺ (*n* = 3, orange), and NR_DTH⁻ (*n* = 4, red) groups. *P* values calculated using Kruskal–Wallis test with Dunn’s multiple comparisons correction. **G** Scatter plots comparing the frequency of selected intra-vaccine subpopulations (LAMP3⁺ DCs, CD8⁺ T cells, CD4⁺ T cells, B cells, NK cells) identified by scRNA-seq between the two patient groups. *P* value determined by two-sided Mann–Whitney *U* test. **H** Frequency of CD19⁺ B cells among live cells in DCVax products, assessed by flow cytometry, in Rs (*n* = 7, blue), NR_DTH⁺ (*n* = 3, orange), and NR_DTH⁻ (*n* = 6, red) groups. *P* values calculated using Kruskal–Wallis test with Dunn’s multiple comparisons correction. Data are presented as mean ± S.E.M.; each dot represents one patient. **I** Side-by-side comparison of t-SNE maps and flow cytometry dot plots from a representative R (P1) and NR (P9), illustrating B cell frequency within the DCVax products. DTH, delayed-type hypersensitivity

DCVax were manufactured under GMP conditions, following the protocol described by Ridolfi and Colleagues [21]. As schematically illustrated in Fig. 1A, monocytes were isolated from leukapheresis products by adherence to culture flasks (2 h) and subsequently cultured in CellGro DC medium (Cell Genix) supplemented with interleukin-4 (IL-4) and granulocyte–macrophage colony-stimulating factor (GM-CSF) which were added at the start of the culture (day 0) and replenished during medium change (day 5). On day 6, 90% of the immature DCs were pulsed with autologous tumor lysate (ATL) or autologous tumor homogenate (ATH) at a concentration of 100 µg/mL, while the remaining 10% were pulsed with keyhole limpet hemocyanin (KLH, 50 µg/mL) as an immune monitoring antigen. On day 7, the medium was replaced, and DCs were matured over an additional 48 h using a cocktail of cytokines comprising tumor necrosis factor-α (TNF-α), interleukin-1β (IL-1β), interleukin-6 (IL-6) (all from Cell Genix), and prostaglandin E2 (PGE2, Pfizer). On day 9, mature DCs (median yield: 10.7 × 10⁶ cells; range: 2.2–20.8 × 10⁶) were harvested, washed, resuspended in sterile saline, and administered intradermally. The release criteria and product batch parameters are extensively described by Granato and Colleagues [22]. Each patient received 10⁷ mature DCs intradermally at the base of the thigh or groin every two weeks for four doses, followed by monthly administrations until disease progression, deterioration of clinical condition (ECOG > 2), or depletion of the available tumor lysate. For patients with residual resectable lesions, vaccination was resumed using lysate derived from newly excised tumors.

### Single-cell preparation and sequencing

DCs were thawed in RPMI supplemented with 20% fetal bovine serum (FBS), 1% DNase I (Sigma-Aldrich, Cat D5025), and counted. To eliminate dead cells and debris, the Dead Cell Removal Kit (Cat 130—090—101, Miltenyi Biotec) was used according to the manufacturer's instructions. The flow-through was subsequently re-counted and resuspended at a final concentration of 1,000 cells/μL in PBS supplemented with 0.4% bovine serum albumin (BSA; Sigma-Aldrich, Cat. A2153—100G). A total of eight DCVax samples from patients were initially selected for scRNA-seq based on the availability of sufficient residual DCVax material after clinical use, adequate cell quality and viability, and fulfillment of technical requirements for single-cell library preparation. One sample was subsequently excluded due to insufficient cell viability. The final dataset comprised three responders (R) and four non-responders (NR; all NR_DTH⁻). Single-cell libraries were prepared using the Chromium Single Cell 5' Library & Gel Bead Kit v1.1 (10 × Genomics, CA, USA, Cat FC51000167). Cells and uniquely barcoded gel beads were loaded onto the Chromium Next GEM Chip K (10 × Genomics, CA, USA) and partitioned into nanoliter-scale Gel Beads-in-Emulsion (GEMs). Inside each GEM, cells underwent lysis and reverse transcription of the released mRNA, which was then barcoded and amplified via 14 cycles of PCR. Barcoded complementary DNAs (cDNAs) longer than 300 bp were purified using 0.6 × SPRIselect beads (Beckman Coulter, Cat B23318), followed by fragmentation, end-repair, and poly(A) tailing. Sample indices were then ligated and libraries amplified. Final libraries were assessed for quality and subsequently sequenced on an Illumina NovaSeq 6000 system (Illumina, San Diego, CA, USA) using 150 bp paired-end reads.

### Single-cell transcriptome data processing and analysis

A total of 7 scRNA-seq libraries were generated and sequenced. Detailed data quality metrics are provided in Supplementary Table 2. Raw sequencing data were processed using Cell Ranger v4.0.0 (10 × Genomics, CA, USA), which involved alignment to the GRCh38 human reference genome, barcode and unique molecular identifier counting, and generation of the digital gene expression matrix. Low-quality cells and potential doublets were excluded using the Seurat R package (v4.3.0.1). For downstream analysis, mature DCs were identified by LAMP3 expression, and clustering of the remaining cells was performed using the Seurat R package (v4.3.0.1). Cell-type annotation was further refined using the *clustifyr* R package (v1.5.1), leveraging reference-based classification to assign cell identities.

### BCR mapping from scRNA-seq data

Immune repertoires from scRNA-seq data were extracted using MiXCR v4.6.0 (MiLaboratories Inc, USA) [23], a universal framework that processes big immunome data from raw sequences to quantitated clonotypes. VDJviz software [24], a web-based graphical user interface application, was used to browse and analyze the immune repertoire sequencing data. Heavy (IgH), Light Kappa (IgLκ) and Lambda (IgLλ) CDR3 sequences have been examined separately. VDJviz data processing provides the number of total and clonotype-specific reads, clonotype diversity, defined as the total number of unique clonotypes, mean clonotype frequency, CDR3 nucleotide/aminoacidic sequences and other BCR repertoire metrics. The donut chart quantile plots to visualize the repertoire clonality were generated excluding singletons and doubletons (clones represented by a single or double reads, respectively) from the CDR3 clonotype frequency rank and subgroup the remaining clonotypes in 5 groups (Q1-Q5; 20% each).

### Multiparametric flow cytometry

Peripheral blood mononuclear cells (PBMCs) from the apheresis product used as starting material for the DCVax preparation were collected and cryopreserved. Samples were thawed in RPMI 1640 supplemented with 10% FBS and 1% 125 U DNase I, washed in autoMACS running buffer and stained for 10 min at 4 °C in the dark with the antibodies listed in Supplementary Table 3. Appropriate conjugated REA Controls (S) were included for each sample. The Inside Stain Kit (Miltenyi Biotec) was used for intracellular staining. Briefly, after surface antigen staining, cells were incubated with Inside Fix for 20 min at room temperature in the dark, then washed and incubated with Inside Perm and intracellular antibody for 10 min at 4 °C in the dark. Samples were washed twice in the autoMACS running buffer before acquisition. Dead cells were excluded from the analysis using 7-amino-actinomycin D (7-AAD) staining solution according to the manufacturer’s instructions. Flow cytometry was performed using a MACSQuant Analyzer 10 (Miltenyi Biotec, RRID: SCR_020268) equipped with 405 nm (violet), 488 nm (blue), and 640 nm (red) lasers. A total of at least 10,000 events were recorded per sample. The acquisition and analysis gates were defined based on forward (FSC) and side scatter (SSC) properties to identify lymphocytes, monocytes, or DCs. Both FSC and SSC were measured on a linear scale. The gating strategy used for subset identification is provided in Supplementary Fig. 1. Data were analysed using the FlowJo Software v10.9.

### CD14⁺ monocyte isolation, purity assessment, and RNA extraction

CD14⁺ monocytes were isolated from apheresis products (*n* = 20) or PBMCs of Healthy Donors (HD) (*n* = 7) (Supplementary Table 1) using positive magnetic selection (CD14 MicroBeads, Cat. 130—050—201, Miltenyi Biotec) following the manufacturer’s protocol. A fraction of the magnetically isolated cells was used to assess purity by flow cytometry. 50,000 cells were stained with anti-CD14 FITC and anti-CD3 APC antibodies for 15 min at room temperature in the dark (Supplementary Table 3). Approximately 10,000 events were acquired using the Attune NxT Flow Cytometer (Thermo Fisher Scientific). An unstained control sample was included for gating reference (Supplementary Fig. 1). Total RNA was extracted from purified CD14⁺ monocytes using the RNeasy Mini Kit (QIAGEN), including on-column DNase I treatment to eliminate genomic DNA contamination. RNA quantity and integrity were evaluated prior to library preparation using the Qubit High Sensitivity RNA Assay Kit (Thermo Fisher Scientific) and the Agilent RNA 6000 Pico Kit (Agilent Technologies).

### Bulk RNA sequencing

RNA libraries were prepared using the Illumina Stranded Total RNA Prep with Ribo-Zero Plus kit (Illumina, San Diego, CA, USA), following the manufacturer's protocol, starting from 100 ng of total RNA. The quality of the final libraries was assessed using the 2100 Bioanalyzer system with the DNA 1000 Kit (Agilent, Santa Clara, USA) and concentrations were determined with the Qubit dsDNA BR Assay Kit (Thermo Fisher Scientific, Waltham, Massachusetts, USA). Libraries were sequenced in paired-end mode (2 × 150 cycles) on the Illumina NextSeq550 (Illumina, San Diego, CA, USA). Transcript-level read count was performed with kallisto v0.46.2, then raw counts were collapsed to gene-level with tximport v1.12.1, then Differential Expression Analysis was performed with DESeq2 v1.22.1121–123. The same software was used to produce PCA plots, while for heatmaps and hierarchical clustering Seaborn v0.12.1 was used. Gene Set Enrichment Analysis (GSEA) was performed with the package GSEA v4.3.3. For Quality Control analyses, DESeq2’s Variance Stabilizing Transformation (VST) was applied to raw read counts, and the 500 genes with the highest variance across the dataset were selected to perform PCA and hierarchical clustering. Sample purity, as inferred from RNA-seq data, exceeded 90% in all cases except for four samples. This estimation was supported by CIBERSORTx (https://cibersortx.stanford.edu) deconvolution analysis, which confirmed their predominant classification within the myeloid compartment. To mitigate potential confounding effects from contaminating cell populations, we generated a list of genes identified by CIBERSORTx as likely not belonging to the myeloid compartment (Supplementary Table 4). These genes were excluded from downstream differential expression analyses used to quantify the number of upregulated and downregulated genes across the four clinical groups: HD, Rs, NR_DTH⁻, and NR_DTH⁺. As expected, the most substantial transcriptomic differences were observed between HD and the three patient groups, in terms of the number of differentially expressed genes (Supplementary Fig. 2).

### Immunohistochemistry (IHC) stainings

Pre-treatment tumor samples were collected either from lesions surgically excised for the preparation of ATL or ATH, or from a prior surgical excision performed within the preceding year. Tissue samples obtained through surgery were fixed in neutral buffered formalin and embedded in paraffin. Three-micron sections were mounted on positive-charged slides (Bio Optica, Milan, Italy). Immunostaining for CD3, CD20 and CD21 was performed using the Ventana Benchmark ULTRA staining system (Ventana Medical Systems, Tucson, AZ, USA) with the Optiview DAB Detection kit (Ventana Medical Systems) and the UltraView Universal Alkaline Phosphatase Red Detection kit (Ventana Medical Systems) for double and single immunostaining, respectively. All the tissue sections were counterstained with Hematoxylin II (Ventana Medical System) after each immunostaining.

### Image acquisition and TLS assessment

High-resolution whole-slide images (40x) of immunohistochemically stained sections were acquired using the Aperio CS2 slide scanner (Leica Biosystems Nussloch GmbH). All double CD20-CD3 IHC stainings were independently reviewed and scored by a trained pathologist (F.L.) based on a four-tiered classification system for TLS. For preparing illustration DAB&RED stainings were converted to pseudo-color images using ImageJ Colour Deconvolution plugin, for stain unmixing. Tissues samples were assigned to one of four categories (Cat#0–3). Cat#0 denoted absence of B cells; Cat#1 indicated presence of B cells without structured aggregates or with aggregates with less than 50 immune cells. Cat#2 samples included organized B and T cell lymphoid aggregates, consistent with immature TLS architecture (iTLS), whereas Cat#3 indicated the presence of mature TLS (mTLS), defined by at least one structure containing CD21⁺ FDCs within a germinal center-like region-hallmarks of advanced structural and immunological organization. All Categories were defined considering the criteria that B cells should be surrounded by tumor cells and/or embedded in the tumor stroma, as well as distant from the residual lymphoid parenchyma when evaluated within lymphoid metastases. It is noteworthy that Cat#3 does not always translate into higher B cell abundance but rather into the compartmentalization of B cells in specific nests.

### Statistical analysis

Statistical analyses were performed using GraphPad Prism v10.4.2 (GraphPad Software, Inc.) or R version 4.4.2. Continuous variables were reported as mean ± standard error of the mean (S.E.M.) or as median, minimum, maximum, and quantiles, depending on data distribution. Categorical variables were presented as frequencies and percentages. For comparisons involving three or more groups, nonparametric data were analyzed using Kruskal–Wallis tests followed by Dunn’s post hoc tests for pairwise comparisons, whereas parametric data were analyzed using one-way ANOVA followed by Tukey’s post hoc tests. For analyses involving two groups, nonparametric data were analyzed using Mann–Whitney U tests, and parametric data using Student’s t-tests. Paired tests were performed in cases where measurements were taken from the same subjects under different conditions. Correlations between continuous variables were assessed using Spearman’s rank correlation coefficient. Statistical tests applied are provided in each figure legend. *P* values < 0.05 were considered statistically significant.

## Results

### Study design

In this study, we evaluated 21 patients with late-stage melanoma. An overview of patient characteristics and the applied technological approaches is provided in Table 1, Supplementary Table 1 and Fig. 1A. Patients were stratified by Best Overall Response (BOR) into Responders (Rs, *n* = 8), including one Complete Response (CR), three Partial Responses (PR), and four patients with Stable Disease (SD), and Non-Responders (NRs, *n* = 13) exhibiting Progressive Disease (PD). A further distinction was made based on DTH testing after at least four vaccinations. While all Rs were DTH +, a subset of NRs exhibited a positive DTH response (NR_DTH⁺, *n* = 4 vs NR_DTH^−^, *n* = 9), indicating the presence of vaccine-induced immunoreactivity. None of the patients had previously received ICI therapy; all had experienced disease progression following systemic treatments before enrollment in the vaccine protocol.

### B-cell abundance in DCVax is associated with a favorable clinical outcome

CD80, CD83, CD86, and HLA-DR are routinely assessed as part of GMP-compliant quality control and batch release criteria at our facility. As previously reported [22], our quality control data consistently confirm high expression of maturation markers, with cell viability and purity exceeding 90% and 60%, respectively, throughout the manufacturing process. Notably, none of these parameters has shown a significant association with clinical outcome (Supplementary Fig. 3 A and B). Similarly, the expression of inhibitory immune checkpoints such as PD-L1, PD-L2, TIM-3, B7-H3, and VISTA, although expressed, did not differ between Rs and NRs (Supplementary Fig. 3 C). Thus, to investigate the cellular and molecular heterogeneity of the DCVax product, we performed scRNA-seq on seven final products, three derived from Rs and four from NRs (Fig. 1A). We obtained high-quality transcriptomic data from 68,329 cells (average of 9,761 per sample), with an average sequencing depth of 53,483 reads per cell, and a mean of 1,664 genes detected per cell (Supplementary Table 2). After quality control, 65,991 cells were retained for downstream analysis, including clustering and neighborhood detection. Fig. 1B shows the aggregated t-distributed Stochastic Neighbor Embedding (t-SNE) projection across all samples. We initially observed that conventional cell classification algorithms designed for unmanipulated ex vivo immune cells were not optimal for in vitro-generated moDCs, likely due to the altered transcriptomic landscape induced by in vitro differentiation and antigen loading. To address this, we used the *LAMP3* gene (also known as DC-LAMP/CD208), a late-stage maturation marker stably expressed in moDCs according to longitudinal bulk RNA-seq data by Jin P and Colleagues [25], to annotate bona fide mature moDCs. Remaining *LAMP3*⁻ cells were annotated using the *clustifyr* R package (v1.5.1) (Fig. 1C). At the protein level, we confirmed LAMP3 expression by flow cytometry and observed its co-expression with canonical maturation markers CD80, CD86, and HLA-DR (Fig. 1D), validating its use as a maturation indicator in our cohort. As shown in the scatter plots (Fig. 1E), no statistically significant difference was detected in the frequency of LAMP3⁺ moDCs between Rs and NRs. This result was further corroborated via flow cytometry, adding nine DCVax products, and a similar non-significant trend was observed in the Geometric Mean Fluorescence Intensity (GeoMFI) of LAMP3 among live DCs (Fig. 1F). Conversely, among the non-DC immune cells identified in the final product, including CD8⁺ T cells, CD4⁺ T cells, B cells and Natural Killer (NK) cells, only B cells were found to be significantly enriched in Rs compared to NRs (*p* = 0.0286; Fig. 1G). This result was further corroborated via flow cytometry (*p* = 0.0398; Fig. 1H and I). Moreover, multiple comparison analyses showed that the frequency of B cells in the DCVax product was significantly higher in DTH + compared to DTH- (median 10.15% vs. 2.65%, *p* = 0.017), while no statistically significant associations were found for any of the variables considered (Supplementary Table 5, first column).

### Plasmablast features and B-cell receptor (BCR) diversity define Rs

**Fig. 2:**
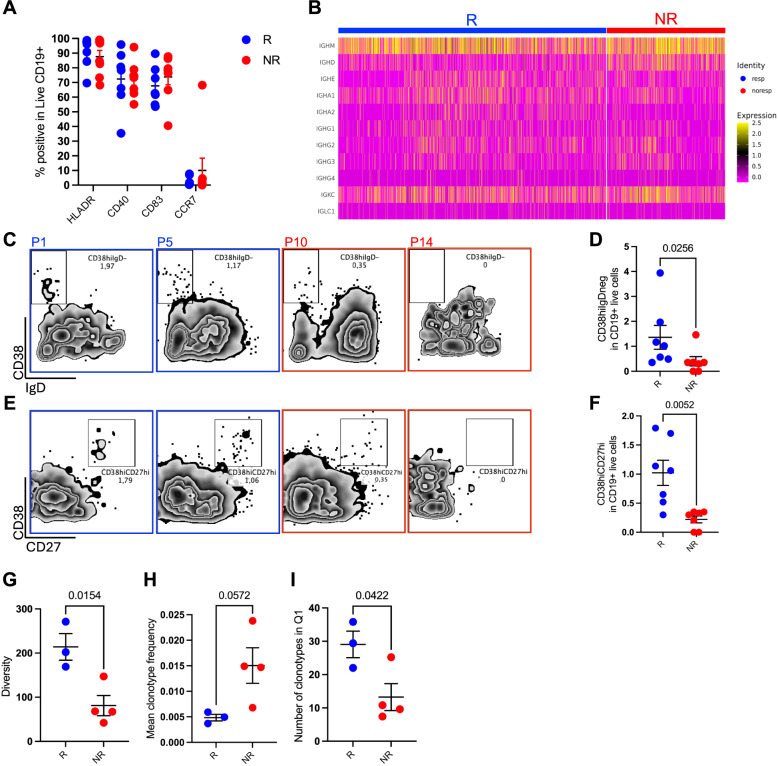
Figure 2. Phenotypic and molecular characterization of intra-DCVax B cells. (A) Expression of co-stimulatory (CD40, CD83), antigen presentation (HLA-DR), and chemokine receptor (CCR7) markers in gated live CD19⁺ B cells from DCVax products. (B) Supervised heatmap showing the abundance of class-switched immunoglobulin gene transcripts at single-cell resolution in R vs. NR samples. (C) Representative dot plot of CD38 vs. IgD expression in CD19⁺ B cells with DCVax products, used to identify class-switched memory B cells. (D) Frequency of CD38^hi^IgD⁻ B cells across analyzed samples (n = 16). P value determined using two-sided Mann–Whitney U test. (E) Representative dot plot of CD38 vs. CD27 expression in CD19⁺ DCVax B cells, highlighting CD38^hi^CD27^hi^ subsets. (F) Frequency of CD38^hi^CD27^hi^ B cells across all samples (n = 16). P value calculated using two-sided Mann–Whitney U test. (G-I) Comparison of B cell receptor (BCR) repertoire metrics between R and NR groups: repertoire diversity (G), mean clonotype frequency (H), and number of clonotypes within the top 20th percentile (Q1) (I) of IgH-CDR3 sequences. P value calculated using a two-tailed unpaired t-test. Data are presented as mean ± s.e.m. Each dot represents an individual patient

To investigate the relevance of our findings, we further characterized the CD19^+^ B cells within the final DCVax formulation. These B cells were also positive for canonical antigen presentation and co-stimulatory markers (HLA-DR, CD40, CD83), albeit with no significant differences between Rs and NRs (Fig. 2A). Single-cell transcriptomic analysis of immunoglobulin genes (e.g., *IGHM*, *IGHD*, *IGHE*, *IGHA*, *IGHG*) suggested that NRs had a higher proportion of IgD⁺ B cells. Conversely, Rs exhibited more class-switched Ig isotypes, reinforcing the notion of B cell maturation in Rs (Fig. 2B). Further flow cytometry analysis using IgD/CD27/CD38 markers indeed demonstrated significantly higher frequencies of IgD⁻CD38^high^ (*p* = 0.0256) and CD38^high^CD27^high^ (*p* = 0.0052) B cells in Rs (Fig. 2C-F). Finally, BCR immune repertoire analysis was performed on scRNA-seq data (*n* = 7) using MiXCR [26] and VDJviz software [27]. IgH-CDR3 sequences from Rs (*n* = 3) showed a significantly higher BCR clonotype diversity (214.0 ± 52.05) compared to NRs (*n* = 4) (81.0 ± 45.61) (*p* = 0.0154; Fig. 2G), and, accordingly, a lower mean clonotype frequency (0.0048 ± 0.0011 vs 0.015 ± 0.07) (*p* = 0.0572; Fig. 2H). When analyzing the top 20% clonotypes (Q1 quantile), Rs exhibited a higher number of unique clonotypes (29.07 ± 6.91) than NRs (13.25 ± 8.09) (*p* = 0.0422; Fig. 2I), indicating a broader distribution of BCR sequences in Rs. These findings were consistent when analyzing IgLκ (Immunoglobulin Light Chain Kappa) and IgLλ (Immunoglobulin Light Chain Lambda) CDR3 sequences separately (Supplementary Fig. 4 A and B, respectively). Characterization of BCR clonality using donut chart quantile plots indicates that 81—94% of counts (except for P14) fall in the top 20% Q1 plot, but with a remarkable difference in terms of both numbers and frequency of clonotypes between Rs and NRs (Supplementary Fig. 4 C and D). Comparing the top 10 most frequent clonotypes in Q1 plot, Rs showed a broader and homogeneous distribution with respect to NR samples, where a few high-frequency clonotypes characterize the Q1 plot. Together, these data demonstrate that DCVax from Rs was enriched in phenotypically and clonally mature B cells, exhibiting plasmablast-like features, activation signatures, and a broad antibody repertoire.

### Pre-treatment enrichment of peripheral B cells, not plasmablast-like cells, in Rs

**Fig. 3:**
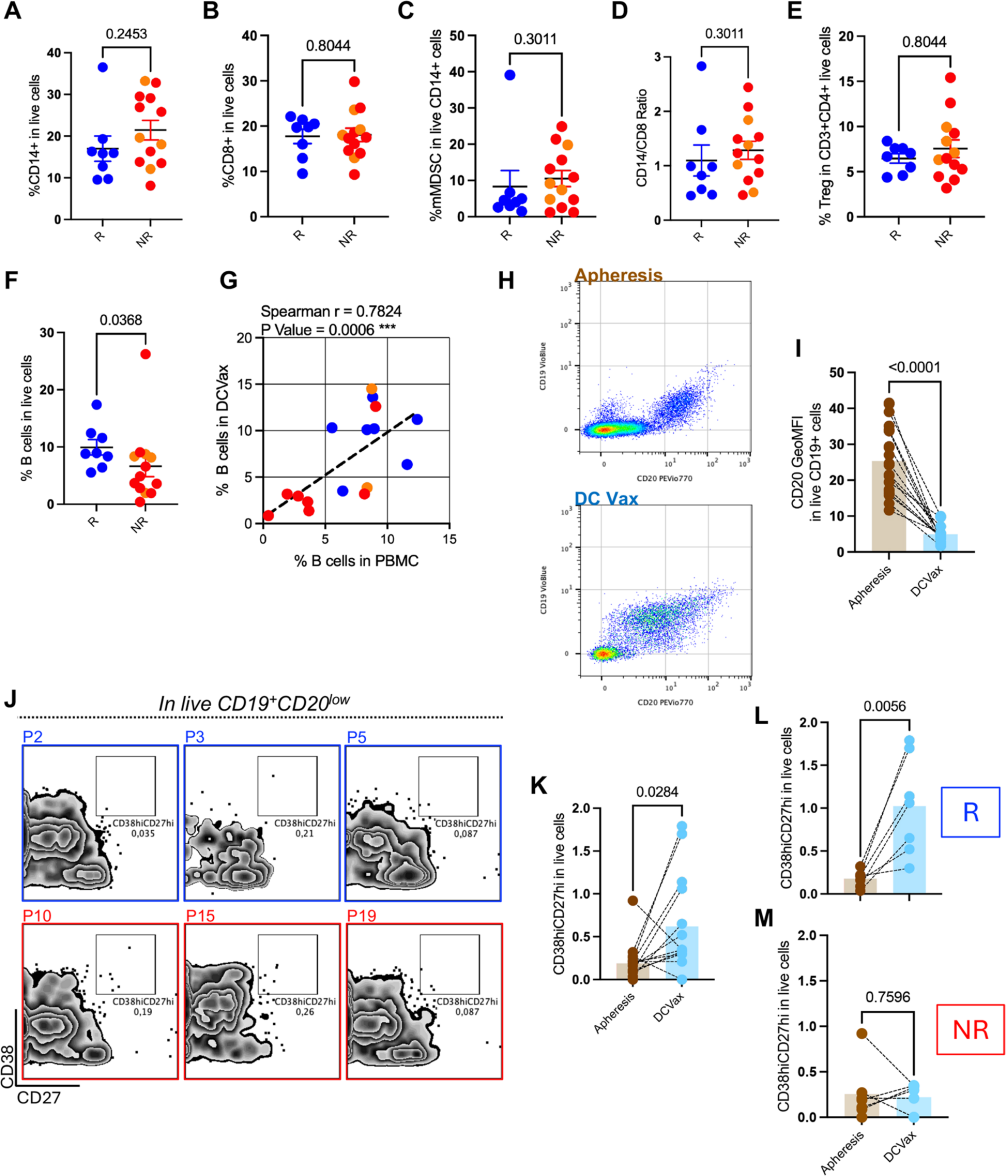
Immune profiling of apheresis samples from DCVax patients. **A-F** Frequency of major immune subsets among live PBMCs: (**A**) CD14⁺ monocytes, **B** CD8⁺ T cells, **C** monocytic myeloid-derived suppressor cells (mMDSCs; HLA-DR^low/neg^ within CD14⁺), **D** CD8/CD14 ratio, **E** CD4⁺CD25^hi^CD127^low^ regulatory T cells (Tregs), and (**F**) CD19⁺CD20⁺ B cells. Each dot represents one patient. *P* values were determined using two-sided Mann–Whitney U test. Data are presented as mean ± S.E.M. **G** Correlation between the frequency of CD19⁺ B cells in DCVax and their corresponding peripheral blood frequency in apheresis samples (*n* = 16). Correlations were assessed using Spearman’s rank test; *r* and *P* values are indicated. Clinical groups are color-coded: Rs (blue), NR_DTH⁺ (orange), and NR_DTH⁻ (red). **H** Representative histograms and dot plots showing CD20 expression within CD19⁺ B cells, comparing matched apheresis samples and final DCVax products. **I** Paired analysis of CD20 GeoMFI in CD19⁺ B cells from apheresis vs DCVax products (*n* = 14). **J** Representative flow cytometry dot plot showing CD38 *vs.* CD27 expression in CD19⁺CD20^low^ B cells from the apheresis product. **K-M** Paired comparison of CD38^hi^CD27^hi^ B cell frequencies between apheresis and DCVax products in all patients (**K**, *n* = 14), in Rs (**L**, *n* = 7), and in NRs (**M**, *n* = 7; includes two NR_DTH⁺). *P* value calculated using two-tailed paired Student’s *t*-test

As shown above, DCVax preparations can include up to 30–40% non-DC cells, mainly B, T, and NK cells, yet only B cells displayed a statistically significant enrichment in Rs compared to NRs. To further explore this, we retrospectively analyzed the apheresis products by multiparametric flow cytometry, profiling major immune subsets in the blood before treatment. We evaluated total CD14⁺ monocytes (Fig. 3A and Supplementary Fig. 2), CD8⁺ T cells (Fig. 3B), monocytic MDSCs (HLA-DR^neg/low^CD14⁺; Fig. 3C), the CD8/CD14 ratio (Fig. 3D), CD4⁺ regulatory T cells (CD4^+^CD25^hi^CD127^low^; Fig. 3E), and B cells (CD19⁺CD20⁺; Fig. 3F).

Consistent with the DCVax findings, only B cells were significantly more abundant in Rs compared to NRs (*p* = 0.0368; Fig. 3F). Multiple comparison testing confirmed this difference (median 8.89% vs. 4.87%, *p* = 0.0357), while no associations were found between peripheral B-cell frequency and clinical or demographic variables (Supplementary Table 5, second column). As expected, B-cell abundance in DCVax strongly correlated with peripheral B-cell levels (*p* = 0.0006; Fig. 3G). Paired comparison of GeoMFI values for CD20 between apheresis and DCVax samples revealed that CD20 expression was significantly lower in CD19⁺ vaccine-infiltrating B cells compared to autologous peripheral blood B cells. (*p* < 0.0001; Fig. 3H and I), confirming a de novo differentiation toward the above-described antibody-producing/plasmablast-like phenotype. Notably, CD20^low^CD27^hi^CD38^hi^ cells were absent from peripheral blood, suggesting that indeed their differentiation occurred within the vaccine product (Fig. 3J). Of note, these populations were enriched in DCVax compared to paired peripheral blood samples (Fig. 3K), only in Rs (Fig. 3L), but not in NRs (Fig. 3M).

### Increase of CD20ˡᵒʷCD38^hi^CD27^hi^ B cells and downregulation of Fc receptors during DCVax manufacturing

**Fig. 4:**
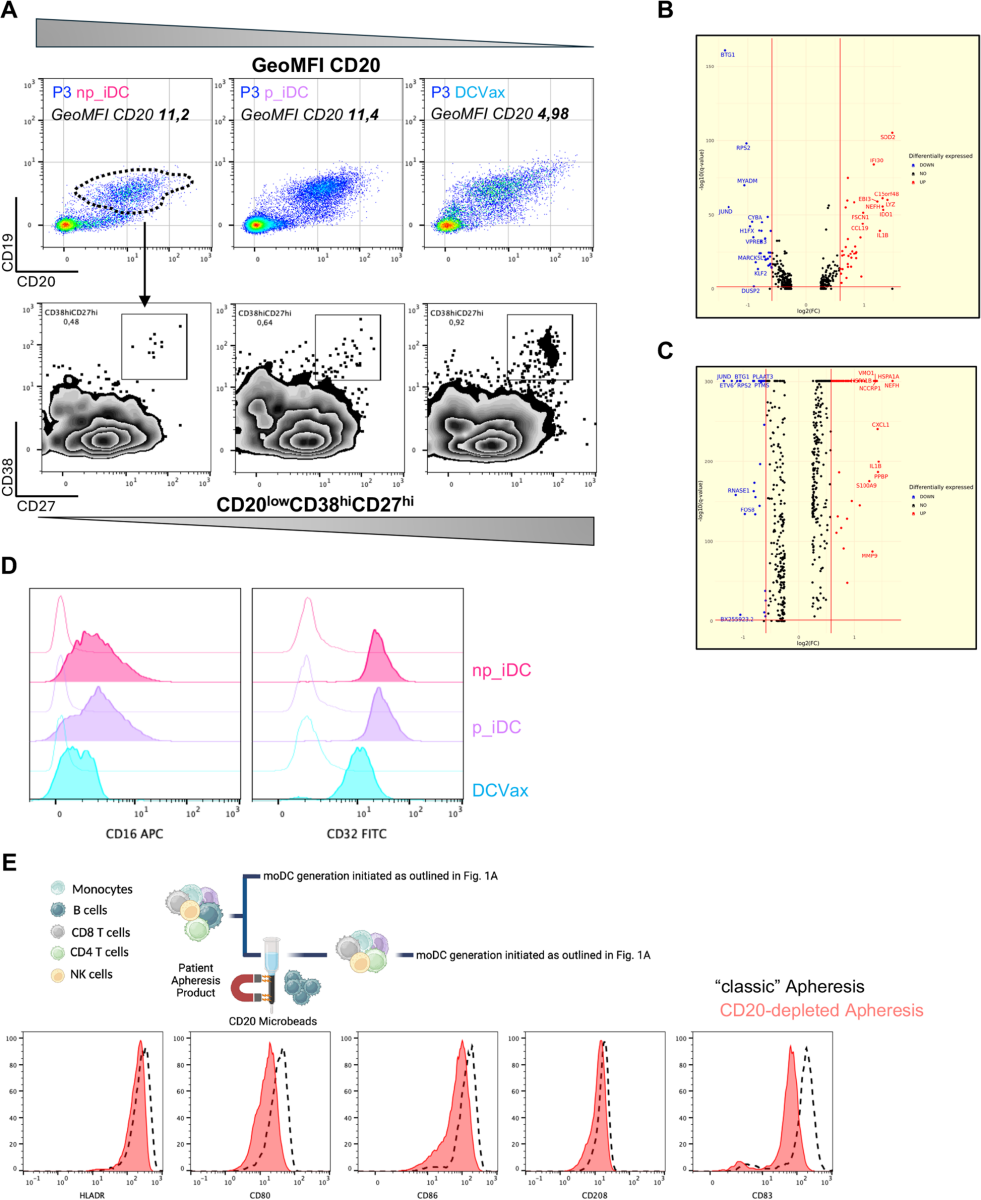
Longitudinal dynamics of CD19⁺CD20^low^CD38^hi^CD27^hi^ B cells and Fc receptor expression during DCVax manufacturing.** A** Representative flow cytometry plots showing the expression of CD19 *vs.* CD20 (top) and CD38 *vs.* CD27 (bottom) in B cells across DCVax maturation stages: non-pulsed immature DCs (np_iDC), pulsed immature DCs (p_iDC), and DCVax. **B** Volcano plot of differentially expressed genes (DEGs) in CD19⁺ DCs comparing R *vs.* NR. **C** Volcano plot of DEGs in LAMP3⁺ DCs comparing R *vs.* NR. Red dots represent significantly upregulated genes, and blue dots represent significantly downregulated genes based on an adjusted *p*-value ≤ 0.05 and log₂ fold change ≥ log₂ (1.5)(*P* < 0.05; log₂ fold change ≥ 0.25). Statistical significance was determined using the Wilcoxon Rank Sum test, and *p*-values were adjusted for multiple testing using the Bonferroni correction. **D** Overlay histograms showing expression of Fc receptors, FcγRIII (CD16, left), FcγRII (CD32, middle), on live cells at the three DCVax stages. Filled histograms represent specific signals from np_iDC, p_iDC, and DCVax, while empty histograms indicate fluorescence-matched isotype controls. **E** Schematic of the workflow for untouched CD19⁺ B cell depletion from the apheresis product of a R patient (P3), created with BioRender. Flow cytometry histograms showing the expression of maturation markers used for GMP release (HLA-DR, CD80, CD83, CD86), and LAMP3 in DCs generated using the standard (“classic”) protocol (dotted lines) versus after CD19⁺ B cell depletion (filled red histograms)

Using multiparametric flow cytometry, we monitored the evolution of CD20^low^CD38^hi^CD27^hi^ B cell subpopulation in intermediate DCVax products, namely non-pulsed immature DCs (np_iDC) and antigen-pulsed immature DCs (p_iDC) (Fig. 1A), obtained from a R patient. As confirmation of previous finding, progressive decrease in CD20 GeoMFI (from 15 to 5) was observed, alongside an increase in the frequency of CD38^hi^CD27^hi^ B cells (from 0.21% to 0.92%), consistent with a differentiation trajectory toward a plasmablast-like phenotype (Fig. 4A). Several other genes, *SOD2*, *IFI30*, *LYZ*, *IDO1*, *IL1B*, were significantly enirched in the B cell cluster of Rs compared to NRs, suggesting a unique activated state in these B cells (Fig. 4B). Notably, differential gene expression analysis of LAMP3⁺ DC clusters from Rs revealed an upregulation of *HSPA1A* and *HSPA1B*, encoding stress-inducible members of the HSP70 family (Fig. 4C). These molecular chaperones are known to facilitate antigen processing and enhance DC activation. Based on literature evidence, HSP70 proteins may act downstream of Fcγ receptor (FcγR) engagement with soluble antigens derived from tumor lysates, promoting the delivery of antigenic peptides to MHC class I loading compartments and thereby boosting cross-presentation efficiency [23, 28]. To explore this possibility, we investigated whether the expression of FcγR family members, namely CD16 (FcγRIII) and CD32 (FcγRII), undergoes modulation during monocyte-to-DC differentiation. Interestingly, both Fc receptors were detectable in both non-pulsed and pulsed iDC stages. Still, their expression was markedly reduced in the final DCVax product (Fig. 4D), consistent with receptor engagement or differentiation-dependent downregulation. Consistently, B-cell depletion performed on leftover apheresis material from a R patient (P3) resulted in detectable alterations in the final moDC phenotype. In particular, the expression levels of the three key costimulatory molecules (CD80, CD86, and CD83) were reduced, indicating that B-cell coexistence contributes to shaping DC differentiation during DCVax manufacturing (Fig. 4E).

### FcR characterizes monocytes of responders

**Fig. 5:**
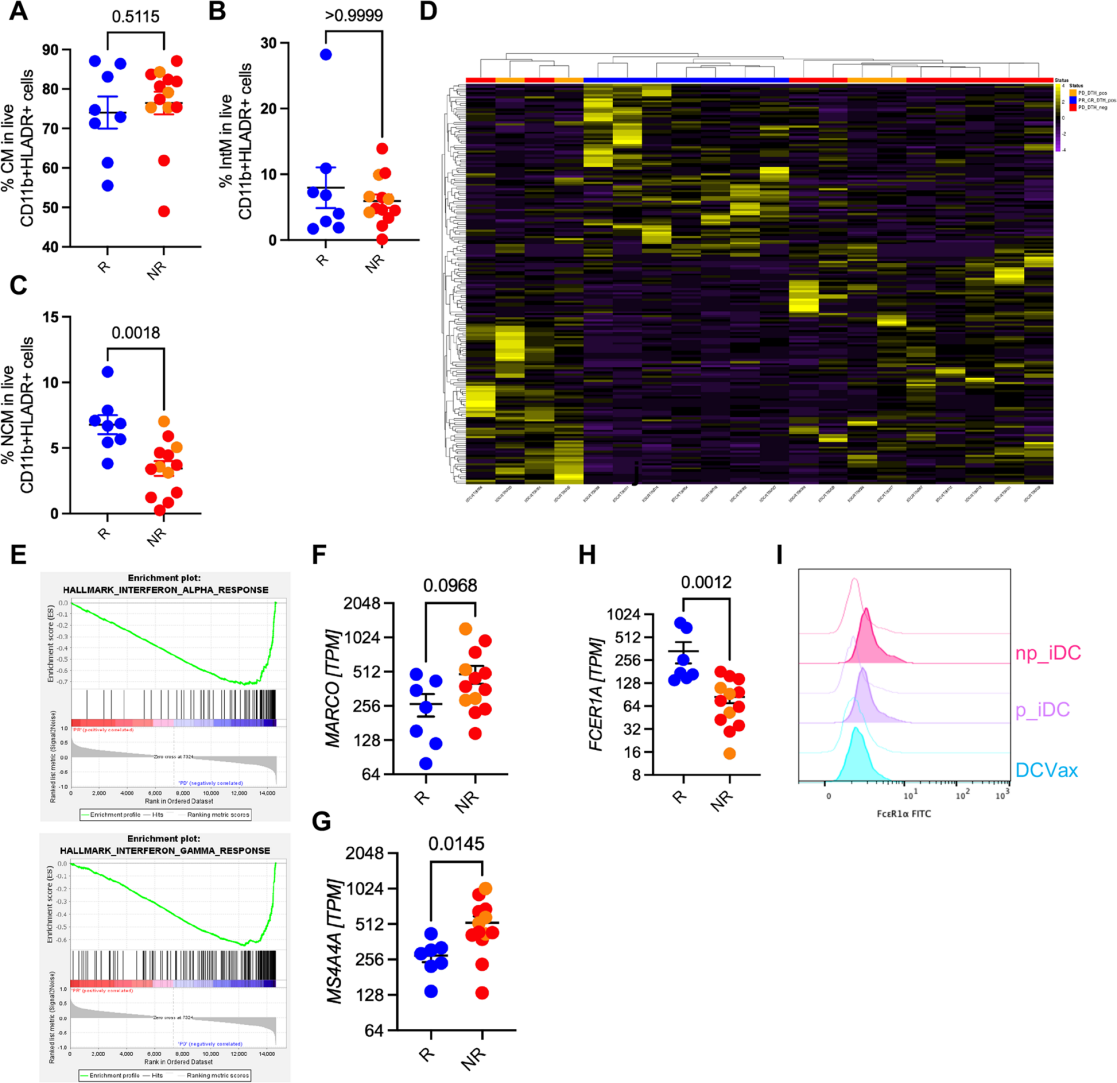
Phenotypic and molecular analysis of DCVax precursor monocytes. **A-C** Scatter plots showing the percentage of the three circulating monocyte subsets: **A** classical (CM; CD14⁺CD16⁻), **B** intermediate (IntM; CD14⁺CD16⁺), and (**C**) non-classical (NCM; CD14dimCD16⁺). **D** Heatmap showing unsupervised clustering based on normalized mRNA expression levels of differentially expressed genes (DEGs) among patient groups. **E** Gene set enrichment analysis (GSEA) for IFN-α and IFN-γ pathways, which were the only significantly enriched signatures when comparing Rs *vs.* NRs (NR_DTH⁺ orange, NR_DTH⁻ red). **F**, **G** Scatter plot showing transcript-per-million (TPM) values of *MARCO* and *MS4A4A*. **H** Scatter plot showing TPM values of *FCER1A*. **I** Overlay histograms showing expression of FcεRI on live cells at the three DCVax stages. Filled histograms represent specific signals from np_iDC, p_iDC, and DCVax, while empty histograms indicate fluorescence-matched isotype controls. Each dot represents one patient. *P* value are determined by two-sided Mann–Whitney *U* test. Data are presented as mean ± S.E.M

Building on previous findings of FcR expression in iDCs, and considering that circulating monocytes serve as the precursors of our DCVax, we investigated CD16 expression on peripheral monocytes, a key marker distinguishing classical (CM; CD14⁺CD16⁻), intermediate (IntM; CD14⁺CD16⁺), and non-classical (NCM; CD14dimCD16⁺) subsets. NCMs were significantly enriched in Rs compared to NRs (*p* = 0.0018; Fig. 5A, B and C), a pattern consistently observed across all comparisons and independent of DTH status (median 6.78% *vs*. 3.39%, *p* = 0.003; Supplementary Table 5, third column). Similarly, DTH⁺ patients displayed higher NCM frequencies than DTH⁻ patients (median 6.18% *vs*. 2.95%, *p* = 0.0036; Supplementary Table 5, third column). Although CM proportions did not differ between groups, transcriptomic profiling of CD14⁺ monocytes was performed to identify potential molecular differences between Rs and NRs. The heatmap illustrates unsupervised clustering based on normalized mRNA expression levels of differentially expressed genes (DEGs) across patients (Fig. 5D). While not forming sharply distinct clusters, Rs and NRs exhibited partially segregating transcriptional patterns, with Rs showing a relative downregulation of type I/II interferon–response pathways (IFN-α, IFN-γ) compared with NRs (Fig. 5E). This finding is consistent with prior evidence that prolonged IFN-γ exposure can skew monocytes toward macrophage-like rather than DC-like differentiation [24, 29]. Supporting this, CM from NRs upregulated *MS4A4A* and *MARCO*, genes linked to reduced antigen presentation and impaired T-cell priming (Fig. 5F, G). Conversely, Rs showed increased expression of *FCER1A* within CD14⁺ monocytes, consistent with enhanced Fc receptor signaling potential (Fig. 5H). Similar to CD16 and CD32, a progressive downregulation of the FcεRI was confirmed by flow cytometry across the transition from immature to mature DCs (Fig. 5I). Taken together, these findings confirm FcR-associated traits as distinguishing features of Rs.

### Mature Tertiary Lymphoid Structures (mTLS) are enriched in Rs before treatment

**Fig. 6:**
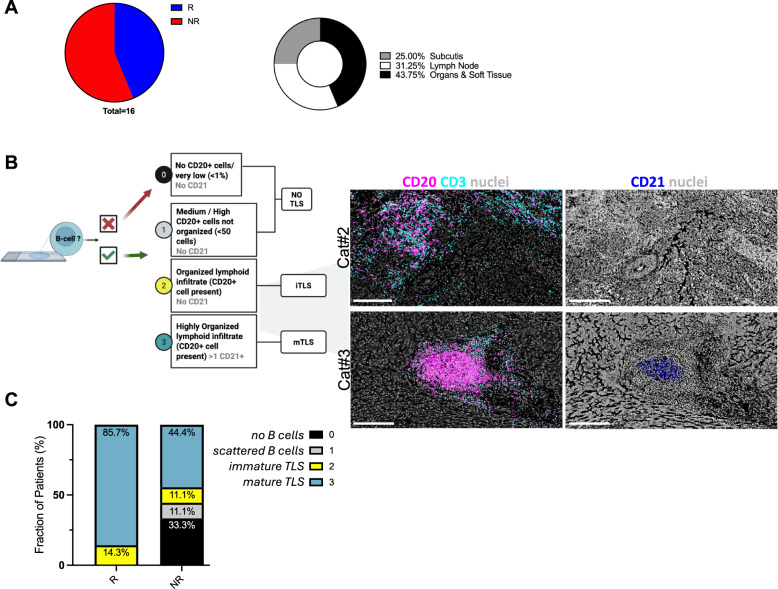
In situ TLS analysis in pre-treatment melanoma samples from DCVax patients. **A** 3 μm FFPE sections were analyzed from a cohort of 16 pre-treatment samples from DCVax patients (7 Rs and 9 NRs). Sample distribution: lymph node metastases (*n* = 5, 31.25%), cutaneous/subcutaneous lesions (*n* = 4, 25.00%), and visceral and soft tissue metastases (*n* = 7, 43.75%). **B** Schematic of the proposed four-tier TLS classification system (Cat#0–3), created with BioRender. Representative pseudo-fluorescence images generated by color deconvolution of DAB (CD20)/RED (CD3) and (RED) CD21 chromogenic IHC stainings, illustrating examples of Cat#2, and Cat#3 TLS. Classification between Cat#2 *vs.* Cat#3 was based on CD21 positivity within the germinal center. Scale bar: 200 μm. **C** Proportion of patients with absent TLS (Cat#0—1), presence of immature TLS (iTLS; Cat#2), or mature TLS (mTLS; Cat#3) within the R and NR groups

Based on the plasmablast-like signature of intra DCVax B cells, we next examined pre-treatment tissue samples to determine whether similar features could be present within the tumor microenvironment. We therefore analyzed 16 pre-treatment FFPE melanoma lesions (7 Rs, 9 NRs; Fig. 6A), including lymph node, cutaneous/subcutaneous, and visceral metastases. Specifically, in light of the B-cell features uncovered earlier, we searched for TLS using CD3/CD20 dual staining, with CD21 as a marker of follicular DCs to assess TLS maturity. Samples were scored from Cat#0–3 (Fig. 6B and Supplementary Table 6). Remarkably, mature TLS (Cat#3) were significantly enriched in Rs, 85.7% versus 44.4% in NRs, while none of the Rs displayed TLS-negative lesions (Cat#0–1), which predominated among NRs (Fig. 6C). Interestingly, two out of five NR patients with Cat#2–3 TLS were DTH⁺. Together, these findings reveal a consistent enrichment of peripheral B cells and MTLS in Rs, supporting a role for organized B-cell responses in shaping a favorable immune baseline that enhances DCVax efficacy.

## Discussion

Despite the broad use, particularly in the past, of tumor-antigen–pulsed moDCs in cancer vaccination, clinical outcomes remain variable and unpredictable. Leveraging these cells could be of paramount importance given their well-tolerated safety profile in immunotherapy combination regimens. Using retrospective samples from our internal cohort, we investigated features associated with clinical benefit by single-cell transcriptomics of GMP-grade moDCs. Although this technology has been applied to other infused ATMPs, notably CAR-T cells [30], this represents, to our knowledge, the first application to manufactured moDCs, offering high-resolution insights into the final product. The major observation was the significant representation of B cells within the DCVax product in Rs. Beyond their increased abundance, a CD20^low^CD27^hi^CD38^hi^ subpopulation occurred within these B cells, indicative of ongoing differentiation of antibody-producing cells. We acknowledge the lack of a fully mature phenotype, which is arguably expected, given the artificial in vitro culture conditions in the absence of exogenous B cell-specific cytokines [31]. Notably, this subset was absent in the circulating B cell compartment of both Rs and NRs, indicating that differentiation is occurring within the vaccine product itself. Based on preclinical findings demonstrating a bidirectional regulation upon DC-B cell interaction [32, 33], these cell types may mutually influence their functional commitment. Furthermore, analysis of the BCR repertoire revealed significantly higher clonotypic diversity in Rs, suggesting an enhanced capacity to recognize a broad spectrum of tumor-associated antigens. This polyclonal response is likely shaped by the complex antigenic landscape of the tumor lysate used for moDC loading, and may underlie the observed clinical benefit, mirroring reports linking intratumoral B-cell diversity to favorable outcomes in cancer immunotherapy [7, 34]. Based on the present findings, it is reasonable to speculate that the upregulation of stress-inducible HSP70 family members observed in the LAMP3⁺ DC cluster of Rs may reflect antibody-related processes in plasmablast-like B cells, potentially involving antigen binding and immune complex (IC) formation during manufacturing. This is consistent with evidence implicating endoplasmic reticulum stress pathways in the cross-presentation of antigen–antibody complexes [35], which my contribute to the observed differences in clinical outcome between Rs and NRs. These findings suggest that plasmablast-like B cells may represent functionally relevant components of DCVax immunobiology, such as antigen uptake and presentation. Several lines of evidence, both in humans and mice, have convincingly demonstrated that ICs promote superior downstream antigen processing and presentation through the binding to Fc receptors, compared to the delivery of “naked” antigen [23, 36–39]. Based on our FcR expression analysis in iDC and circulating monocytes, a potential role for IgE/FcεRI-mediated antigen cross-presentation, beyond CD16 and CD32 can also be envisaged [40]. Moreover, our findings suggest using baseline TLS/B-cell features to stratify candidates and to time DC vaccination when a B-cell–rich microenvironment is already present. This extends the recognized role of TLS, previously defined primarily in the context of ICI therapy, to DC‑vaccinated melanoma patients, indicating that B‑cell activity, both within the tumor microenvironment and in the DCVax product, may significantly influence therapeutic efficacy. Interestingly, a recent study demonstrated the recreation of germinal centres–like structures in vitro by supplying B cells with phagocytic antigen [41]. Although our DCVax platform is not specifically designed to replicate the full architecture of TLS, it appears to recapitulate key cellular interactions found in mTLS-rich tumors. In particular, the co-presence of DCs, antibody-producing B cells, and CD4⁺ T cells, suggests the partial reconstitution of an adaptive immune niche within the vaccine product itself. This may contribute to enhanced antigen presentation and immune priming in patients predisposed to respond. Notably, no significant differences in overall CD4⁺ T-cell frequency were observed between Rs and NRs (Fig. 1G). To further investigate this aspect, we analyzed CD4⁺ T-cell subpopulations, including naïve, central memory, effector memory, regulatory, and helper subsets, using established single-cell annotation frameworks [42]. However, no significant differences emerged across CD4⁺ T-cell subsets (Supplementary Fig. 5). Rather, the most prominent difference between Rs and NRs lies in the increased representation and differentiation state of B cells observed in Rs, supporting their relevance as a key component of the vaccine-associated immune context, whereas other immune populations, although potentially fundamental, do not display significant differences between the two groups. Of note, in situ CD16⁺ macrophages were recently reported to associate with clinical response in pre-treatment tumor tissues from ICI-treated melanoma patients [43]. While the proposed mechanism in that study involves Fc region binding to CD16 on immunostimulatory macrophages, it is equally plausible that the presence of ICs, potentially enriched in mTLS-positive tumors [44], facilitates cross-presentation and supports durable antitumor responses. A similar mechanism may explain our observation that NCM frequency correlates with both clinical benefit and DTH positivity. This study provides a basis for further investigation into how B cell–DC interactions influence the efficacy of DC-based cancer vaccines. The observation of plasmablast differentiation during vaccine manufacturing, coupled with the absence of this phenotype in patients’ circulation, and the concurrent FcR expression and HSP70 upregulation, are not consistent with B cells being passive byproducts. Instead, these features support a potential functional contribution of B cells to vaccine potency, a hypothesis that warrants prospective testing through controlled B-cell depletion or add-back during GMP manufacturing. Consistent with this idea, our preliminary B-cell depletion experiment already revealed alterations in the final moDC phenotype. Addressing current limitations, including the use of retrospectively collected frozen samples and a relatively small but clinically homogeneous cohort, will be crucial to further validate and expand these findings. In this context, although the scRNA-seq analysis was conducted on a limited number of samples, it was designed as an in-depth characterization of representative DCVax products rather than a comprehensive profiling of the entire cohort. Importantly, the observed patterns were consistently supported by orthogonal analyses across a larger set of samples, including flow cytometry and tissue-based evaluations.

## Conclusions

In summary, this study provides a high-resolution characterization of plasmablast-like B-cell populations within GMP-manufactured dendritic-cell vaccines, highlighting their association with clinical outcome. Their differentiation within the vaccine product, together with Fc-receptor expression on DCs and the selective upregulation of HSP70 family members, supports a model in which B cells may contribute to these processes, although a direct functional role cannot be inferred from the present data. The concordance between vaccine-embedded B-cell activity and baseline TLS/B-cell features further indicates that pre-existing B-cell–rich immune contexts may shape clinical responsiveness. Although preliminary and based on a relatively small cohort, these findings may inform future strategies for optimizing GMP manufacturing protocols and for identifying patient-specific features associated with improved response. They also open the way to mechanistic studies, such as controlled B-cell depletion or add-back experiments, to determine the causal contribution of B cells to vaccine potency. Together, our data provide a rationale for further exploration of next-generation DC-vaccine platforms that deliberately incorporate immune-supportive interactions to enhance therapeutic efficacy across solid tumors.

## Supplementary Information

Supplementary Material 1.

Supplementary Material 2.

Supplementary Material 3.

Supplementary Material 4.

Supplementary Material 5.

Supplementary Material 6.

Supplementary Material 7.

## Data Availability

Sequencing data generated in this study have been deposited in the European Genome-phenome Archive (EGA) at: (https://ega-archive.org) under study no. EGAS50000001177 (with a summary of patient characteristics). Data access is controlled and can be granted upon reasonable request and approval by the Data Access Committee.

## Abbreviations

ATL: Autologous Tumor Lysate
ATH: Autologous Tumor Homogenate
ATMP: Advanced Therapy Medicinal Product
BOR: Best Overall Response
BCR: B Cell Receptor
CD: Cluster of Differentiation
cDNAs: complementary DNAs
CM: Classical Monocytes
CR: Complete Response
DC: Dendritic Cell
DCVax: Dendritic Cell Vaccine
DEG: Differentially Expressed Gene
DTH: Delayed-Type Hypersensitivity
FBS: Fetal Bovine Serum
FcR: Fc Receptor
FcγR: Fc Gamma Receptor
FcεRI: High-affinity IgE Receptor
FFPE: Formalin-Fixed Paraffin-Embedded
FSC: Forward Scatter
GEMs: Gel Beads-in-Emulsion
GeoMFI: Geometric Mean Fluorescence Intensity
GMP: Good Manufacturing Practice
GSEA: Gene Set Enrichment Analysis
HD: Healthy Donor
HLA-DR: Human Leukocyte Antigen – DR isotype
HSP70: Heat Shock Protein 70
IC: Immune Complex
ICI: Immune Checkpoint Inhibitor
iDC: Immature Dendritic Cell
iTLS: Immature Tertiary Lymphoid Structure
Ig: Immunoglobulin
IgH: Immunoglobulin Heavy Chain
IgLκ: Immunoglobulin Light Chain Kappa
IgLλ: Immunoglobulin Light Chain Lambda
IL: Interleukin
IntM: Intermediate Monocytes
KLH: Keyhole Limpet Hemocyanin
LAMP3: Lysosomal Associated Membrane Protein 3
moDC: Monocyte-derived Dendritic Cell
mMDSC: Monocytic Myeloid-Derived Suppressor Cell
mTLS: Mature Tertiary Lymphoid Structure
NCM: Non-Classical Monocytes
NK: Natural Killer
np_iDC: Non-pulsed Immature Dendritic Cell
NR: Non-Responder
OS: Overall Survival
PBMC: Peripheral Blood Mononuclear Cell
PCA: Principal Component Analysis
PD: Progressive Disease
PGE2: Prostaglandin E2
PR: Partial Response
R: Responder
RECIST: Response Evaluation Criteria in Solid Tumors
RNA-seq: RNA Sequencing
scRNA-seq: Single-cell RNA Sequencing
SD: Stable Disease
SEM: Standard Error of the Mean
SSC: Side Scatter
t-SNE: T-distributed Stochastic Neighbor Embedding
TLS: Tertiary Lymphoid Structure
TNF-α: Tumor Necrosis Factor Alpha
