# Supplementary figures and images for: Vaccine-expanded plasmablast-like B cells are associated with response to dendritic cell therapy in metastatic melanoma
Source: J Exp Clin Cancer Res. 2026 May 23;45:138. doi: 10.1186/s13046-026-03731-5 (PMC13277274; doi:10.1186/s13046-026-03731-5)

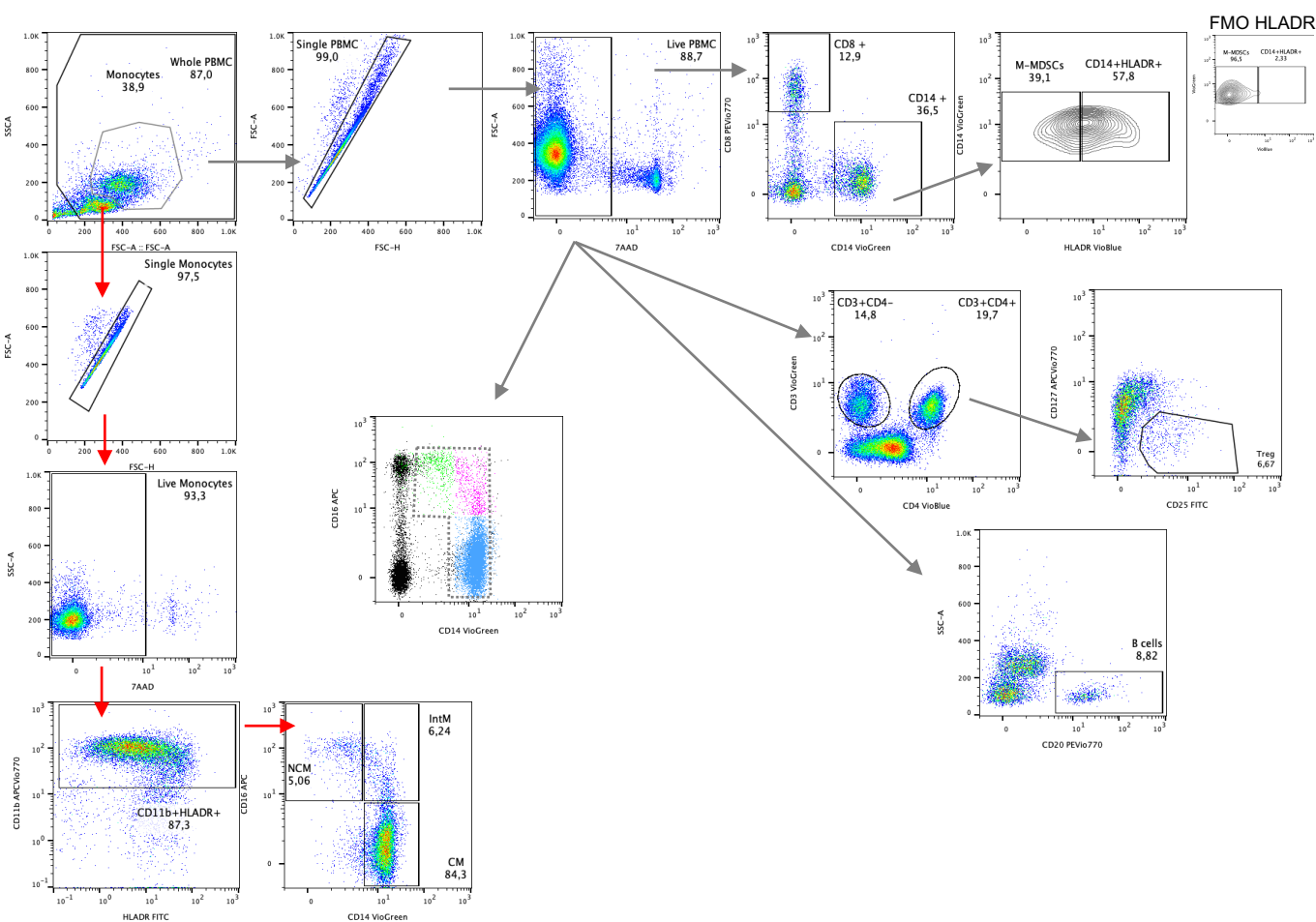

**Suppl. Figure 1**

Supplement: Supplementary file 1 — Supplementary Material 1. [file 13046_2026_3731_MOESM1_ESM.pdf]

**A**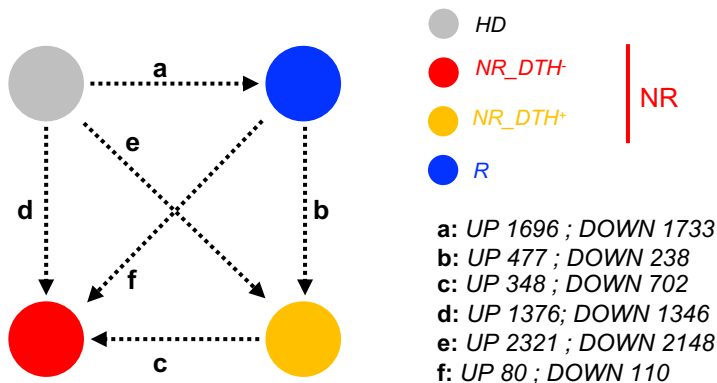**B**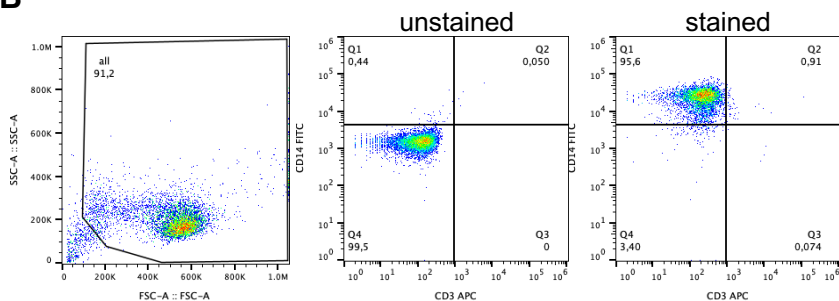**C**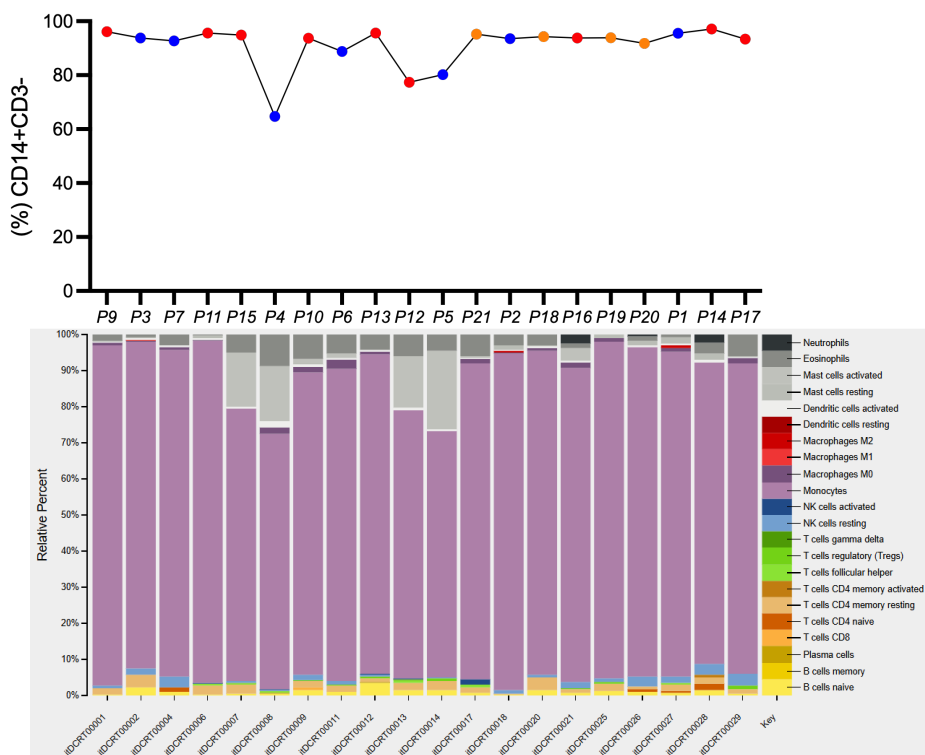**Suppl. Figure 2**

Supplement: Supplementary file 2 — Supplementary Material 2. [file 13046_2026_3731_MOESM2_ESM.pdf]

**A**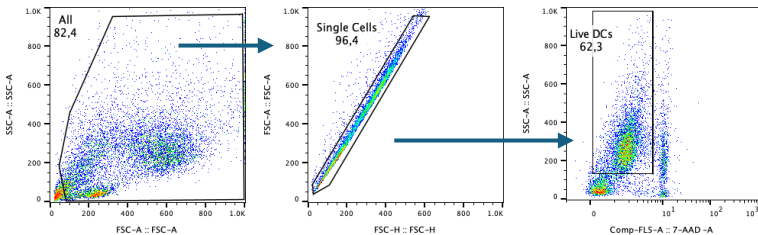**B**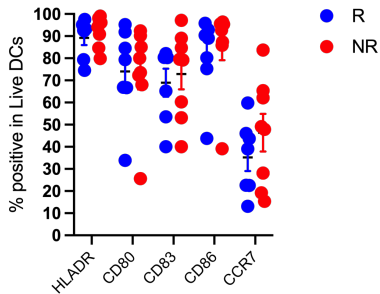**C**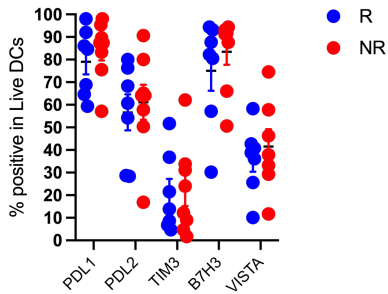**Suppl. Figure 3**

Supplement: Supplementary file 3 — Supplementary Material 3. [file 13046_2026_3731_MOESM3_ESM.pdf]

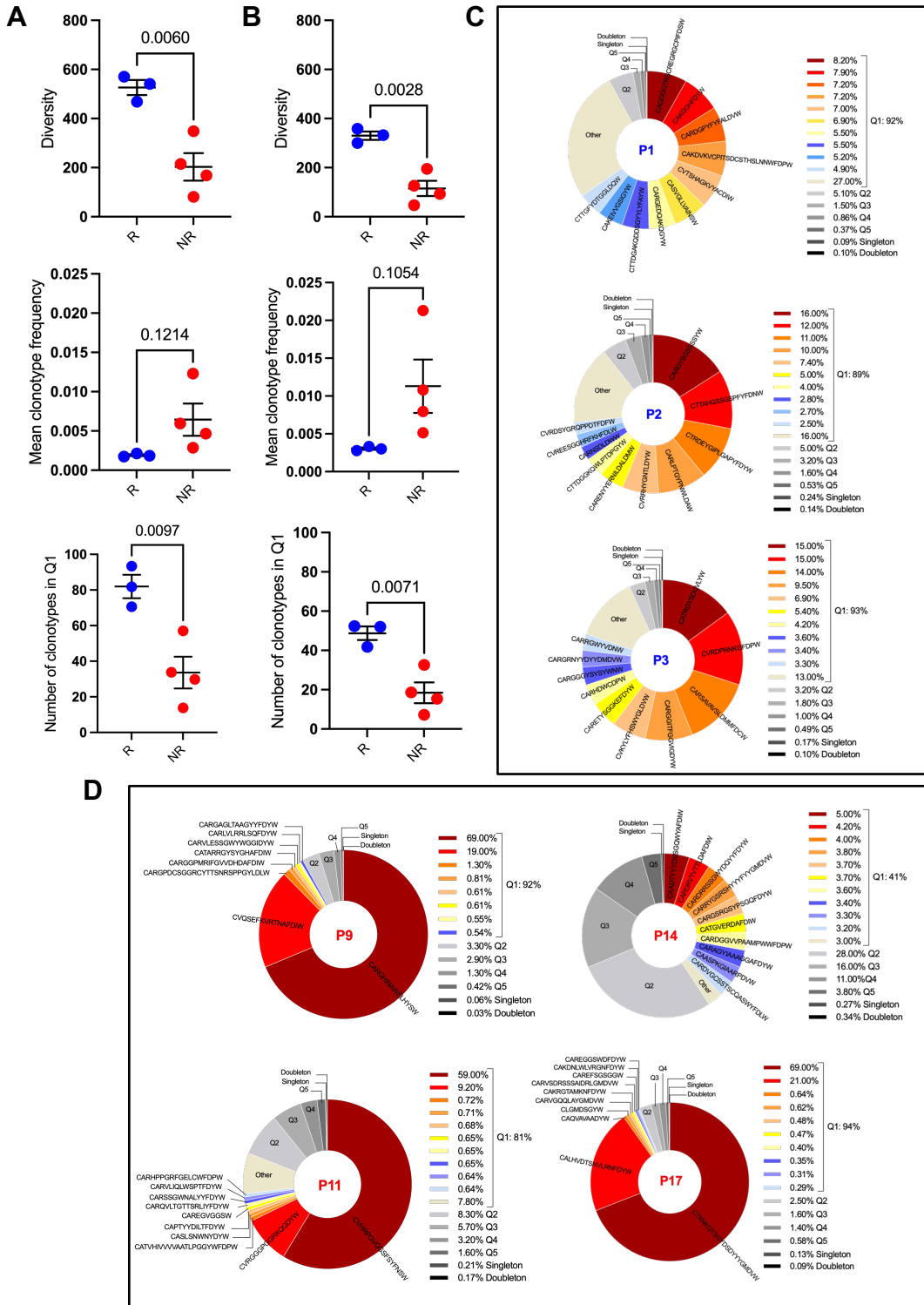

Supplement: Supplementary file 4 — Supplementary Material 4. [file 13046_2026_3731_MOESM4_ESM.pdf]

**A**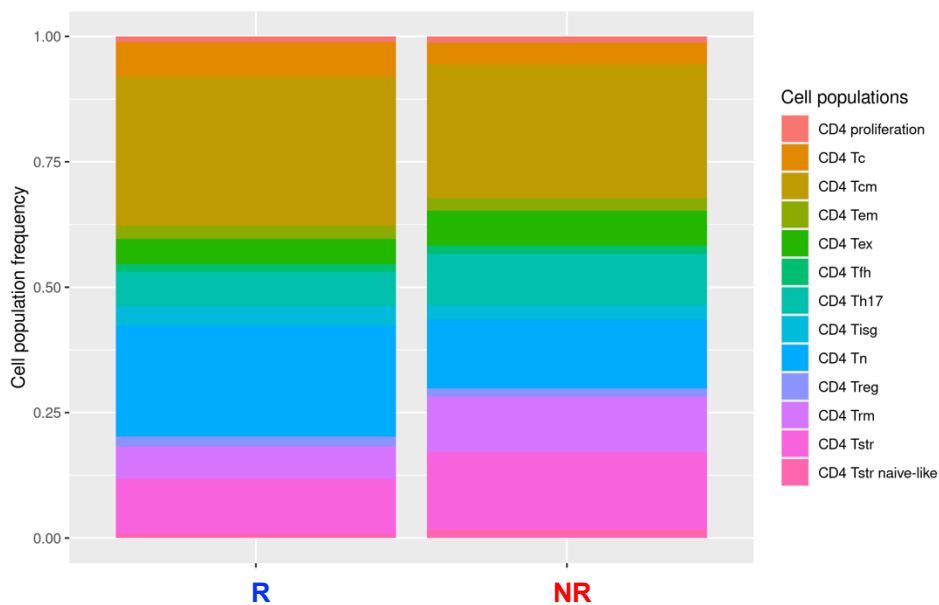**B**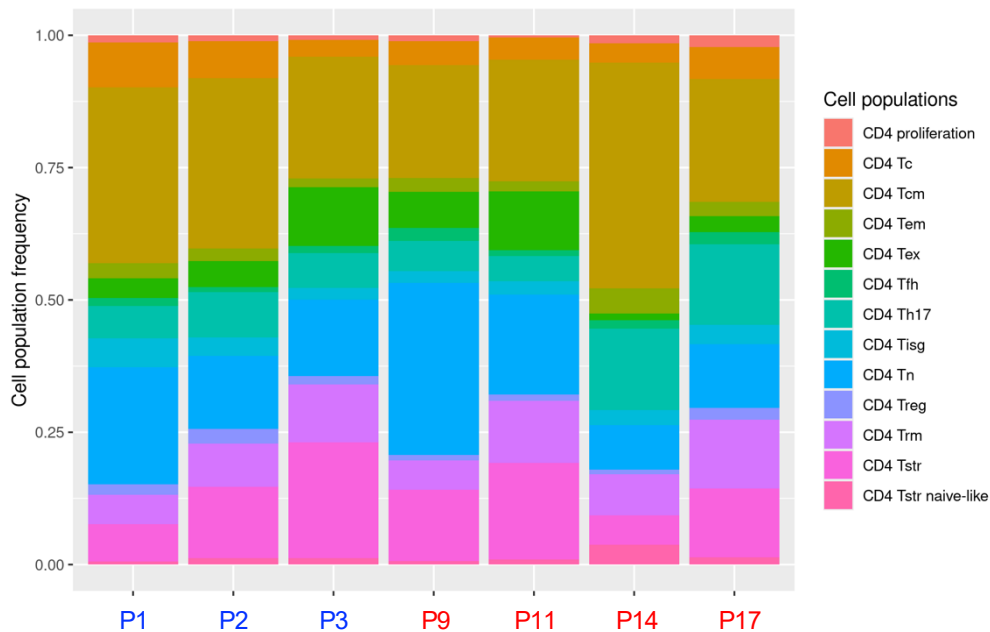

Supplement: Supplementary file 5 — Supplementary Material 5. [file 13046_2026_3731_MOESM5_ESM.pdf]

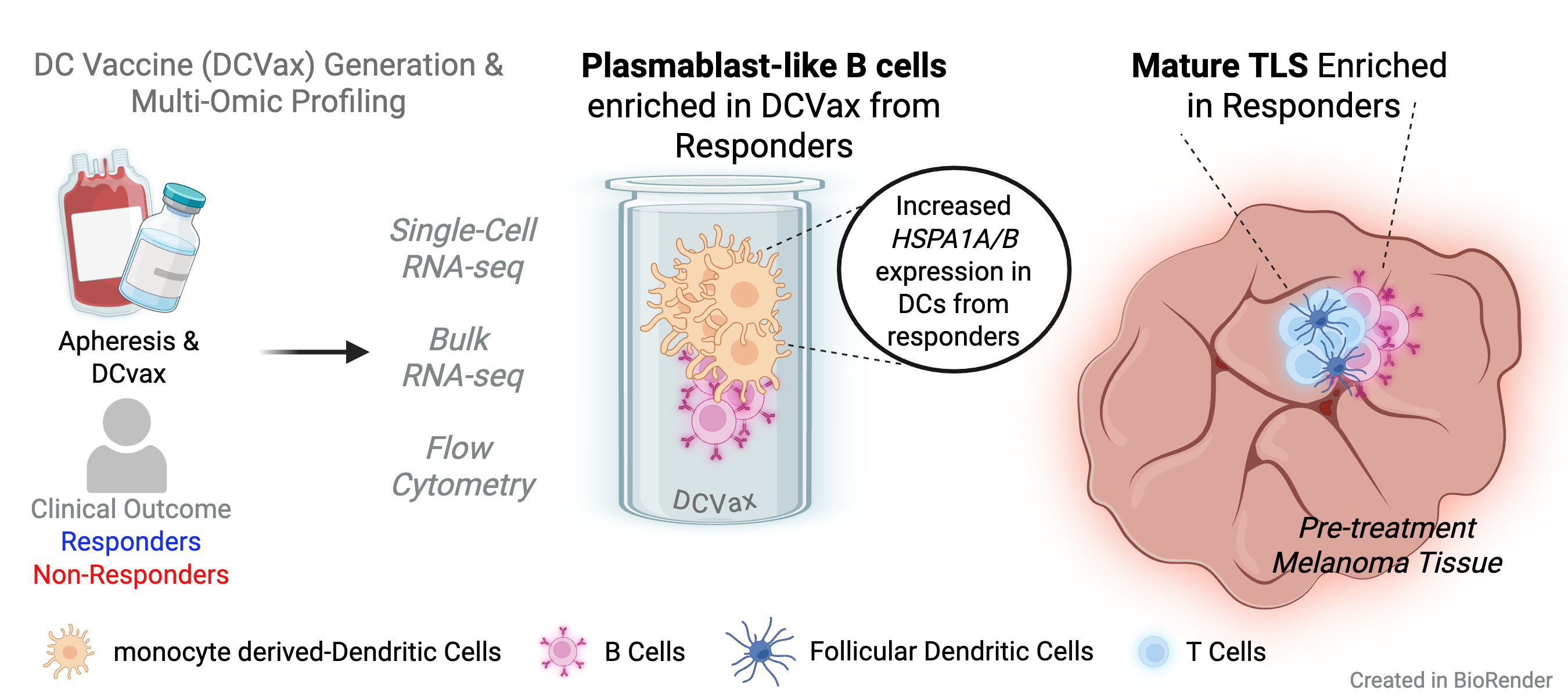

Supplement: Supplementary file 7 — Supplementary Material 7. [file 13046_2026_3731_MOESM7_ESM.png]
