# Supplementary material for: Vaccine-expanded plasmablast-like B cells are associated with response to dendritic cell therapy in metastatic melanoma
Source: J Exp Clin Cancer Res. 2026 May 23;45:138. doi: 10.1186/s13046-026-03731-5 (PMC13277274; doi:10.1186/s13046-026-03731-5)
Supplement: Supplementary file 6 — Supplementary Material 6. [file 13046_2026_3731_MOESM6_ESM.docx]

**Supplementary Informations to**

**Vaccine-Expanded Plasmablast-like B Cells Are Associated with Response to Dendritic Cell Therapy in Metastatic Melanoma**

Marcella Tazzari^1*^, Silvia Carloni^2^, Jenny Bulgarelli^1^, Sara Pignatta^2^, Martine Bocchini^1^, Claudia Piccinini^2^, Davide Angeli^3^, Michela Tebaldi^3^, Irene Azzali^3^, Maria Maddalena Tumedei^1^, Filippo Piccinini^4^, Francesca Tauceri^5^, Francesco Limarzi^6^, Fabio Nicolini^1^, Maria Teresa Bochicchio^7,8^, Milena Urbini^7^, Giovanni Foschi^1^, Nicola Romanini^1^, Francesco de Rosa^1^, Anna Maria Granato^2^, Elena Pancisi^2^, Massimiliano Petrini^2*^, Laura Ridolfi^1^

^1^IRCCS Istituto Romagnolo per lo Studio dei Tumori (IRST) “Dino Amadori”, Meldola (FC), Italy.

^2^Immuno-Gene Therapy Factory, IRCCS Istituto Romagnolo per lo Studio dei Tumori (IRST) "Dino Amadori", Meldola, Italy.

^3^Unit of Biostatistics and Clinical Trials, IRCCS Istituto Romagnolo per lo Studio dei Tumori (IRST) "Dino Amadori", 47014 Meldola, Italy.

^4^Department of Medical and Surgical Sciences (DIMEC), University of Bologna, via G.Massarenti 9, 40138 Bologna, Italy.

^5^General and Oncological Surgery, Morgagni-Pierantoni Hospital, Forlì, Italy.

^6^Pathology Unit, Morgagni-Pierantoni Hospital, AUSL Romagna, 47121, Forlì, Italy.

^7^Biosciences Laboratory, IRCCS Istituto Romagnolo per lo Studio dei Tumori (IRST) “Dino Amadori”, Meldola, Italy.

^8^Unit of Medical Genetics, The Greater Romagna Area Hub Laboratory, Piazza della Liberazione 60, 47522 Cesena, Italy

**Supplementary Table 1. Sample Availability Matrix for Each Technology Used in the Study.**

| **Sample ID** | **Resource** | **Flow Cytometry**  **Apheresis** | **Flow Cytometry**  **DCVax** | **scRNAseq**  **DCVax** | **RNAseq**  **CD14+** | **IHC FFPE** |
| --- | --- | --- | --- | --- | --- | --- |
| P1 | Melanoma Patient | x | x | x | x | x |
| P2 | Melanoma Patient | x | x | x | x | x |
| P3 | Melanoma Patient | x | x | x | x | x |
| P4 | Melanoma Patient | x | x |  | x | x |
| P5 | Melanoma Patient | x | x |  | x | x |
| P6 | Melanoma Patient | x |  |  | x | x |
| P7 | Melanoma Patient | x | x |  | x | x |
| P8 | Melanoma Patient | x | x |  |  |  |
| P9 | Melanoma Patient | x | x | x | x | x |
| P10 | Melanoma Patient | x | x |  | x | x |
| P11 | Melanoma Patient | x | x | x | x | x |
| P12 | Melanoma Patient | x |  |  | x |  |
| P13 | Melanoma Patient | x |  |  | x |  |
| P14 | Melanoma Patient | x | x | x | x |  |
| P15 | Melanoma Patient | x |  |  | x | x |
| P16 | Melanoma Patient | x | x |  | x | x |
| P17 | Melanoma Patient | x | x | x | x | x |
| P18 | Melanoma Patient | x | x |  | x | x |
| P19 | Melanoma Patient | x | x |  | x | x |
| P20 | Melanoma Patient | x | x |  | x | x |
| P21 | Melanoma Patient | x |  |  | x |  |
| HD1 | Healthy Donor | x |  |  | x |  |
| HD2 | Healthy Donor | x |  |  | x |  |
| HD3 | Healthy Donor | x |  |  | x |  |
| HD4 | Healthy Donor | x |  |  | x |  |
| HD5 | Healthy Donor | x |  |  | x |  |
| HD6 | Healthy Donor | x |  |  | x |  |
| HD7 | Healthy Donor |  |  |  | x |  |
| *Abbreviations:* HD, Healthy Donor | | | | | | |

**Supplementary Table 2. Summary of Single-Cell RNA-Seq Quality Control Metrics per Sample.**

| **Sample ID** | **Resource** | **Cell Number** | **Cell After QC** | **Mean Reads per Cell** | **Median UMI Counts per Cell** | **Median Genes per Cell** |
| --- | --- | --- | --- | --- | --- | --- |
| P1 | Melanoma Patient, PR | 11892 | 11706 | 45305 | 13776 | 2754 |
| P2 | Melanoma Patient, SD | 9147 | 9076 | 63549 | 5272 | 1725 |
| P3 | Melanoma Patient, SD | 13202 | 12959 | 27803 | 4944 | 1417 |
| P9 | Melanoma Patient, PD | 6307 | 6174 | 91565 | 2975 | 1169 |
| P11 | Melanoma Patient, PD | 9526 | 9369 | 36757 | 4188 | 1411 |
| P14 | Melanoma Patient, PD | 7963 | 6517 | 62079 | 1936 | 755 |
| P17 | Melanoma Patient, PD | 10292 | 10163 | 47321 | 9716 | 2414 |
| *Abbreviations:* CR, Complete Response; PR, Partial Response; SD, Stable Disease; PD, Progressive Disease; QC, Quality Control | | | | | | |

**Supplementary Table 3. Antibody Used in IHC and the Flow Cytometry Analysis.**

| **Name** | **Clone** | **Dilution** | **Cat. No.** | **Company** | **RRID** |
| --- | --- | --- | --- | --- | --- |
| CONFIRM anti-CD3 Primary Antibody | 2GV6 | ready to use | 790-4341 | Roche | AB_2335978 |
| anti-CD20cy Monoclonal Antibody | L26 | 1:200 | - M0755 | Dako | n.a. |
| Anti-CD21 Monoclonal Antibody | EP3093 | ready to use | 760-4438 | Roche | n.a. |
| BV421 Anti-Human VISTA | MIH65.rMAb | 1:20 | 566751 | BD Biosciences | AB_2869848 |
| VioBlue Anti-Human HLA-DR | REA805 | 1:50 | 130-111-794 | Miltenyi Biotec | AB_2652162 |
| VioBlue Anti-Human CD4 | REA623 | 1:50 | 130-114-534 | Miltenyi Biotec | AB_2726691 |
| VioBlue Anti-Human CD19 | REA675 | 1:100 | 130-120-031 | Miltenyi Biotec | AB_2784030 |
| VioGreen Anti-Human CD3 | REA613 | 1:50 | 130-113-704 | Miltenyi Biotec | AB_2726245 |
| VioGreen Anti-Human CD14 | REA599 | 1:50 | 130-110-525 | Miltenyi Biotec | AB_2655057 |
| FITC Anti-Human CD209 | REA617 | 1:50 | 130-119-784 | Miltenyi Biotec | AB_2751841 |
| FITC Anti-Human HLA-DR | REA805 | 1:100 | 130-111-788 | Miltenyi Biotec | AB_2652156 |
| VioBright B515 Anti-Human CD25 | REA570 | 1:50 | 130-113-287 | Miltenyi Biotec | AB_2784092 |
| VioBright FITC Anti-Human CD38 | REA572 | 1:50 | 130-113-433 | Miltenyi Biotec | AB_2726165 |
| VioBright FITC Anti-Human CD4 | REA623 | 1:50 | 130-113-229 | Miltenyi Biotec | AB_2726040 |
| VioBright FITC Anti-Human CD40 | REA733 | 1:50 | 130-110-950 | Miltenyi Biotec | AB_2657997 |
| PE Anti-Human CD197 (CCR7) | REA108 | 1:50 | 130-120-463 | Miltenyi Biotec | AB_2784045 |
| PE Anti-Human CD83 | REA714 | 1:50 | 130-110-561 | Miltenyi Biotec | AB_2659320 |
| PE Anti-Human IgD | REA740 | 1:200 | 130-110-643 | Miltenyi Biotec | AB_2652262 |
| PE Anti-Human CD80 | REA661 | 1:50 | 130-123-253 | Miltenyi Biotec | AB_2802016 |
| PE-Vio770 Anti-Human CD19 | REA675 | 1:50 | 130-113-647 | Miltenyi Biotec | AB_2726200 |
| PE-Vio770 Anti-Human CD20 | REA780 | 1:50 | 130-111-340 | Miltenyi Biotec | AB_2656074 |
| PE-Vio770 Anti-Human CD8 | REA734 | 1:50 | 130-110-680 | Miltenyi Biotec | AB_2659245 |
| PE-Vio770 Anti-Human CD38 | REA572 | 1:50 | 130-113-432 | Miltenyi Biotec | AB_2733228 |
| APC Anti-Human CD3 | UCHT1 | 1:100 | 300412 | BioLegend | AB_314066 |
| APC Anti-Human CD16 | REA423 | 1:50 | 130-113-389 | Miltenyi Biotec | AB_2726149 |
| APC Anti-Human CD83 | REA714 | 1:25 | 130-110-504 | Miltenyi Biotec | AB_2659323 |
| APC Anti-Human CD138 | REA929 | 1:50 | 130-115-480 | Miltenyi Biotec | AB_2857404 |
| APC-Vio770 Anti-Human CD8 | REA734 | 1:50 | 130-110-681 | Miltenyi Biotec | AB_2659247 |
| APC-Vio770 Anti-Human CD11b | REA713 | 1:50 | 130-110-556 | Miltenyi Biotec | AB_2654675 |
| APC-Vio770 Anti-Human CD127 | REA614 | 1:50 | 130-113-416 | Miltenyi Biotec | AB_2726162 |
| APC-Vio770 Anti-Human CD27 | REA449 | 1:50 | 130-113-637 | Miltenyi Biotec | AB_2819368 |
| VioBlue REA Control Antibody (S) | REA293 | - | 130-113-442 | Miltenyi Biotec | AB_2733973 |
| VioGreen REA Control Antibody (S) | REA293 | - | 130-113-444 | Miltenyi Biotec | AB_2734114 |
| FITC REA Control Antibody (S) | REA293 | - | 130-113-437 | Miltenyi Biotec | AB_2733689 |
| VioBright FITC REA Control Antibody (S) | REA293 | - | 130-113-443 | Miltenyi Biotec | AB_2734084 |
| VioBright B515 REA Control Antibody (S) | REA293 | - | 130-113-445 | Miltenyi Biotec | AB_2734052 |
| PE REA Control Antibody (S) | REA293 | - | 130-113-438 | Miltenyi Biotec | AB_2733893 |
| PE-Vio770 REA Control Antibody (S) | REA293 | - | 130-113-440 | Miltenyi Biotec | AB_2733280 |
| APC REA Control Antibody (S) | REA293 | - | 130-113-434 | Miltenyi Biotec | AB_2733447 |
| APC-Vio770 REA Control Antibody (S) | REA293 | - | 130-113-435 | Miltenyi Biotec | AB_2733167 |
| 7-AAD | n.a. | 1:10 | 130-111-568 | Miltenyi Biotec | n.a. |
| *Abbreviations:* n.a., not applicable | | | | | |

**Supplementary Table 4. Selected CIBERSORT Gene List Used in the Bioinformatic Analysis.**

| **B cells memory** | ADAM28, AIM2, ALOX5, BACH2, BANK1, BLK, CCR6, CD180, CD19, CD1C, CD22, CD27, CD37, CD69, CD72, CD79A, CD79B, CLCA3P, CR2, CXCR5, DENND5B, FAIM3, FAM65B, FCGR2B, FCRL2, FRK, GNG7, GPR18, GUSBP11, HHEX, HLA-DOB, IFNA10, IGHD, IGHM, IGKC, IGLL3P, IL7, IRF8, KIAA0226L, LTB, LY86, MBL2, MS4A1, NMBR, NPIPB15, P2RX5, PNOC, PTPRCAP, RALGPS2, RASGRP2, SIK1, SIT1, SLC12A1, SP140, SPIB, STAP1, TMEM156, TNFRSF13B, TNFRSF17, TRAF4, VPREB3, ZBTB32 |
| --- | --- |
| **B cells naive** | ABCB4, ADAM28, BACH2, BANK1, BCL7A, BEND5, BLK, BRAF, CD180, CD19, CD1C, CD22, CD37, CD69, CD72, CD79A, CD79B, CR2, CXCR5, EAF2, FAIM3, FCER2, FCGR2B, FCRL2, FRK, GPR18, GUSBP11, HHEX, HLA-DOB, IGHD, IGHM, IGKC, IGLL3P, IL4R, IRF8, KIAA0226L, LINC00921, LTB, LY86, MEP1A, MICAL3, MS4A1, NIPSNAP3B, NMBR, P2RX5, P2RY14, PNOC, PSG2, PTPRCAP, RALGPS2, RASGRP2, SELL, SIK1, SLC12A1, SPIB, STAP1, TCL1A, UGT1A8, VPREB3, ZNF286A |
| **T cells CD4 memory activated** | CCL20, CD2, CD247, CD28, CD3D, CD3G, CD40LG, CD6, CD7, CDC25A, CSF2, CTLA4, CXCL13, DPP4, GPR171, GPR19, GZMB, ICOS, IFNG, IL12RB2, IL17A, IL26, IL2RA, IL3, IL4, IL9, LAG3, LCK, LTA, NKG7, ORC1, PMCH, RRP9, SH2D1A, SKA1, TNFRSF4, TNIP3, TRAC, TRAT1, UBASH3A |
| **T cells CD4 memory resting** | BCL11B, CCL5, CCR6, CD2, CD247, CD27, CD28, CD3D, CD3E, CD3G, CD4, CD40LG, CD6, CD69, CD7, CD96, CTLA4, CTSW, DGKA, DPP4, EPB41, ETS1, FAIM3, FBXL8, FLT3LG, GPR171, GPR25, GRAP2, GZMA, GZMK, GZMM, ICOS, IL7R, ITK, KLRB1, LCK, LEF1, LIME1, LTB, LY9, NKG7, PBXIP1, PTGER2, PTPRCAP, RASA3, RASGRP2, RCAN3, RPL10L, RPL3P7, SH2D1A, SIRPG, ST8SIA1, TCF7, TRAC, TRAT1, TRAV13-1, TRAV13-2, TRAV21, TRAV8-6, TRAV9-2, TRBC1, UBASH3A, ZAP70, ZFP36L2 |
| **T cells CD4 naive** | ACAP1, ANKRD55, ATHL1, BCL11B, CCR7, CD2, CD247, CD27, CD3D, CD3G, CD40LG, CD7, CXorf57, DPP4, DSC1, EPHA1, FAIM3, FLJ13197, FLT3LG, GAL3ST4, GALR1, GPR1, GRAP2, GZMM, ICOS, IL7R, ITK, LAT, LCK, LEF1, LIME1, LTB, LY9, MAP4K1, MAP4K2, MAP9, RASGRP2, RPL3P7, SERGEF, SH2D1A, SIRPG, TCF7, TRAC, TRAT1, TRAV13-1, TRBC1, UBASH3A, VILL, WNT7A, ZAP70, ZNF204P, ZNF324 |
| **T cells CD8** | BCL11B, CCL5, CD2, CD247, CD27, CD3D, CD3E, CD3G, CD6, CD69, CD7, CD8A, CD8B, CD96, CRTAM, CST7, CTSW, DPP4, DSC1, DUSP2, FAIM3, FLT3LG, GNLY, GPR171, GRAP2, GZMA, GZMB, GZMH, GZMK, GZMM, ICOS, IGKC, IL7R, ITK, KLRB1, KLRC3, KLRC4, KLRD1, KLRF1, KLRK1, LAG3, LCK, LEF1, LIME1, LTB, LY9, MAP4K1, MAP9, NCR3, NKG7, PIK3IP1, PRF1, PTGDR, PTPRCAP, PVRIG, RASA3, RPL3P7, SH2D1A, SIRPG, TCF7, TRAC, TRAT1, TRAV12-2, TRAV13-1, TRBC1, TRDC, UBASH3A, ZAP70 |
| **T cells follicular helper** | ATHL1, BCL11B, CA8, CD2, CD247, CD27, CD3D, CD3G, CD40LG, CD69, CD7, CHI3L2, CTLA4, CXCL13, CXCR5, DGKA, FAIM3, FOSB, FZD3, GPR19, GZMM, ICA1, ICOS, IL21, ITK, KLRB1, LAG3, LAT, LCK, LEF1, LTA, MAP4K1, MAP9, PASK, PDCD1, PTPRCAP, PVRIG, RGS1, RPL3P7, SH2D1A, SIK1, SIRPG, SLC7A10, ST8SIA1, TCF7, TNFRSF4, TRAC, TRAT1, TRAV13-1, TRAV8-6, TRAV9-2, TRBC1, TRIB2, TSHR, UBASH3A, ZAP70, ZBTB10 |
| **T cells gamma delta** | BFSP1, BRSK2, CCL5, CCR5, CD160, CD2, CD244, CD247, CD300A, CD3D, CD3G, CD8A, CD8B, CDH12, COLQ, CST7, CXCR6, DUSP2, GFI1, GNLY, GPR171, GPR18, GYPE, GZMA, GZMB, GZMH, GZMK, GZMM, IL18RAP, IL2RB, KLRB1, KLRD1, KLRG1, KLRK1, KRT18P50, LAG3, LAT, LCK, LHCGR, LY9, MAGEA11, MAP4K1, PLEKHG3, PRF1, PTGDR, PVRIG, SCN9A, SH2D1A, SIRPG, SIT1, SKAP1, TARDBPP1, TCF7, TRBC1, TRDC, UBASH3A, VNN2, ZNF442 |
| **T cells regulatory (Tregs)** | BARX2, BCL11B, CD2, CD247, CD27, CD28, CD3D, CD3E, CD3G, CD4, CD5, CD6, CD70, CD96, CEMP1, CLEC2D, CTLA4, DGKA, DPP4, EFNA5, FOXP3, FRMD8, GPR1, GPR171, GPR19, GZMM, HIC1, HMGB3P30, ICOS, IL2RA, IL2RB, ITK, KIRREL, LAIR2, LCK, LILRA4, LOC126987, LTB, MAP4K1, MBL2, NPAS1, NTN3, PCDHA5, PLCH2, PMCH, PTGIR, PTPRG, RCAN3, RYR1, SEC31B, SEPT5, SH2D1A, SIRPG, SIT1, SKAP1, SPOCK2, SSX1, TRAC, TRAT1, TRAV9-2, TRBC1, TYR, UBASH3A, ZAP70 |
| **NK cells activated** | APOBEC3G, APOL6, CCL4, CCL5, CCND2, CD244, CD247, CD69, CD7, CD96, CDK6, CSF2, CST7, CTSW, DPP4, FASLG, GNLY, GPR171, GPR18, GRAP2, GZMA, GZMB, GZMH, GZMM, IFNG, IL12RB2, IL18R1, IL18RAP, IL2RB, KIR2DL1, KIR2DL4, KIR2DS4, KIR3DL2, KLRB1, KLRC3, KLRD1, KLRF1, KLRK1, LCK, LTA, LTB, NAALADL1, NCR3, NKG7, OSM, PRF1, PRR5L, PTGDR, PTGER2, PTPRCAP, PVRIG, S1PR5, SH2D1A, SOCS1, TBX21, TNFSF14, TRDC, TXK, ZAP70 |
| **NK cells resting** | AZU1, BPI, CAMP, CCL5, CD160, CD2, CD244, CD247, CD7, CD96, CDHR1, CEACAM8, CST7, CTSW, DEFA4, ELANE, GFI1, GNLY, GZMA, GZMB, GZMH, GZMK, GZMM, IL12RB2, IL18R1, IL18RAP, IL2RB, KIR2DL1, KIR3DL2, KLRB1, KLRC3, KLRC4, KLRD1, KLRF1, KLRK1, LCK, MGAM, MS4A3, NAALADL1, NKG7, NME8, PLEKHF1, PRF1, PRR5L, PTGDR, PTPRCAP, PVRIG, S1PR5, SH2D1A, TBX21, TEP1, TRBC1, TRDC, TTC38, TXK, ZAP70, ZNF135 |
| **Plasma cells** | ABCB9, AMPD1, ANGPT4, ATXN8OS, C11orf80, CCR10, CD27, CD38, CD79A, DENND5B, EAF2, FCRL2, GNG7, GPR25, GUSBP11, HIST1H2AE, HIST1H2BG, HLA-DOB, IGHD, IGHE, IGHM, IGKC, IGLL3P, KCNA3, KCNG2, LIME1, LOC100130100, MAN1A1, MANEA, MAST1, MROH7, MZB1, P2RX5, PAX7, PDK1, PNOC, RASGRP3, REN, RGS13, RPL3P7, SIK1, SPAG4, ST6GALNAC4, TGM5, TMEM156, TNFRSF17, UGT2B17, ZBP1, ZNF165 |
| *Note:* Genes highlighted in red were identified as differentially expressed (DEGs) in the CD14⁺ monocyte dataset and subsequently excluded from further analysis, as detailed in the *Materials and Methods* section. | |

**Supplementary Table 5. Results of Multiple Comparisons Between Clinical and Immune Cell Variables.**

|  | **% B cells DC Vax*** | **% B cells Apheresis** | **% NCM Apheresis** |  |  |
| --- | --- | --- | --- | --- | --- |
|  |  |  |  |  |  |
| **BOR**  median, min-max, IQ-IIIQ |  |  |  |  |  |
| R (n=8) | 10.2, 3.51-13.6, 6.35-11.2 | 8.885, 5.55-17.4, 7.385-12 | 6.78, 3.82-10.8, 5.55-7.48 |  |  |
| NR (n=13) | 3.18, 0.86-14.5, 2.34-3.85 | 4.87, 0.42-26.2, 2.82-8.37 | 3.39, 0.25-7.03, 1.61-4.64 |  |  |
| *p* | 0.0640 | **0.0357** | **0.003** |  |  |
|  |  |  |  |  |  |
| **DTH** |  |  |  |  |  |
| median, min-max, IQ-IIIQ |  |  |  |  |  |
| DTH + (n=12) | 10.15, 3.19-14.5, 3.85-11.2 | 8.555, 1.96-17.4, 7.28-10.275 | 6.18, 3.12-10.8, 4.44-7.06 |  |  |
| DTH - (n=9) | 2.65, 0.86-12.6, 1.37-3.18 | 3.68, 0.42-26.2, 2.82-6.6 | 2.95, 0.25-5.9, 1.23-4.45 |  |  |
| *p* | **0.017** | 0.0646 | **0.0036** |  |  |
|  |  |  |  |  |  |
| **Visceral metastases** |  |  |  |  |  |
| median, min-max, IQ-IIIQ |  |  |  |  |  |
| yes (n=13) | 3.35, 1.37-14.5, 3.07-10.7 | 6.6, 1.93-26.2, 3.68-8.95 | 4.45, 0.25-7.03, 3.12-5.67 |  |  |
| no (n=8) | 8.225, 0.86-13.6, 3.095-11.45 | 8.365, 0.42-11.6, 4.565-8.935 | 5.755, 1.61-10.8, 3.26-7.48 |  |  |
| *p* | 0.6744 | 0.9423 | 0.2183 |  |  |
|  |  |  |  |  |  |
| **Tumor burden**° |  |  |  |  |  |
| median, min-max, IQ-IIIQ |  |  |  |  |  |
| high (n=7) | 3.18, 1.37-11.2, 2.34-3.51 | 3.68, 1.93-12.4, 1.96-6.41 | 5.67, 0.25-7.03, 0.84-6.69 |  |  |
| low (n=14) | 10.1, 0.86-14.5, 3.19-12.6 | 8.555, 0.42-26.2, 6.6-9.05 | 4.545, 1.23-10.8, 3.39-6.87 |  |  |
| *p* | 0.1567 | 0.0524 | 0.6015 |  |  |
|  |  |  |  |  |  |
| **Gender** |  |  |  |  |  |
| median, min-max, IQ-IIIQ |  |  |  |  |  |
| F (n=10) | 10.1, 0.86-14.5, 2.96-10.2 | 7.48, 0.42-26.2, 2.82-8.95 | 4.545, 1.23-7.09, 3.39-5.9 |  |  |
| M (n=11) | 3.85, 1.37-13.6, 3.18-11.2 | 8.15, 1.93-12.4, 3.68-9.05 | 5.06, 0.25-10.8, 1.61-6.87 |  |  |
| *p* | 0.9548 | 0.8880 | 0.9439 |  |  |
|  |  |  |  |  |  |
| **Previous treatment** |  |  |  |  |  |
| median, min-max, IQ-IIIQ |  |  |  |  |  |
| yes (n=15) | 3.51, 0.86-12.6, 2.96-10.2 | 6.6, 0.42-17.4, 2.82-8.95 | 4.64, 0.84-7.09, 2.95-5.9 |  |  |
| no (n=6) | 10.3, 1.37-14.5, 6.35-13.6 | 8.78, 3.68-26.2, 5.55-11.6 | 5.66, 0.25-10.8, 3.12-7.87 |  |  |
| *p* | 0.1926 | 0.2129 | 0.4835 |  |  |
|  |  |  |  |  |  |
| **No. lines previous treat** |  |  |  |  |  |
| median, min-max, IQ-IIIQ |  |  |  |  |  |
| 0-1 (n=17) | 8.225, 0.86-14.5, 3.18-11.2 | 8.36, 0.42-26.2, 4.87-9.05 | 5.06, 0.25-10.8, 3.39-6.69 |  |  |
| ≥2 (n=4) | 3.095, 2.34-3.85, 2.34-3.85 | 5.09, 1.96-8.37, 2.77-7.485 | 2.59, 1.23-7.03, 1.42-5.3 |  |  |
| *p* | 0.3408 | 0.2443 | 0.3245 |  |  |
|  |  |  |  |  |  |
| **Age^#^** |  |  |  |  |  |
| median, min-max, IQ-IIIQ |  |  |  |  |  |
| ≤ 51 y (n=12) | 10.15, 2.34-14.5, 3.345-11.95 | 7.48, 1.93-26.2, 4.565-8.885 | 4.94, 0.84-7.78, 2.365-6.95 |  |  |
| > 51 y (n=9) | 3.52, 0.86-12.6, 2.165-8.775 | 8.15, 0.42-12.4, 3.68-9.05 | 4.64, 0.25-10.8, 3.39-5.67 |  |  |
| *p* | 0.2480 | 0.8312 | 0.7223 |  |  |
| *Notes:* * Data missing for 5 patients; ° Defined as the number of metastatic sites: high, ≥2; low <2; #51 is the median; NCM, non-classical monocytes | | | |  |  |

# **Supplementary Table 6. Description of FFPE Metastatic Tumor Lesions Included in the Study Cohort.**

| **Sample ID** | **Tumor Lesion, site** | **B Cell Category** | **BOR** |
| --- | --- | --- | --- |
| P1 | liver | 2 | CR |
| P2 | soft tissue - mesentery | 3 | SD |
| P3 | lymph node | 3 | SD |
| P4 | liver | 3 | PR |
| P5 | lymph node | 3 | CR |
| P6 | stomach | 3 | SD |
| P7 | lymph node | 3 | PR |
| P9 | subcutis | 0 | PD |
| P10 | lymph node | 3 | PD |
| P11 | lymph node | 1 | PD |
| P15 | subcutis | 0 | PD |
| P16 | subcutis | 3 | PD |
| P17 | ileum | 3 | PD |
| P18 | soft tissue - chest | 2 | PD |
| P19 | soft tissue - abdomen | 0 | PD |
| P20 | subcutis | 3 | PD |
| *Abbreviations:* BOR, Best Overall Response; CR, Complete Response; PR, Partial Response; SD, Stable Disease; PD, Progressive Disease. | | | |

S**upplementary Figure Legends**

**Supplementary Figure 1. Flow cytometry gating strategy.** Gating strategy for flow cytometric analysis of leukapheresis samples. Viable cells were selected using 7-AAD exclusion in both whole PBMCs and gated monocytes, following the exclusion of debris and doublets. CD8⁺ and CD14⁺ cell frequencies were determined within the live PBMC gate. mMDSCs were defined as HLA-DR^neg/low^ within the CD14⁺ population. Tregs were identified as CD25^hi^CD127^low^ within the CD3⁺CD4⁺ T cell compartment. B cells were gated based on CD20 positivity among live PBMCs. To avoid contamination by CD16⁺ non-myeloid populations (e.g., NK or CD8⁺ T cells), monocytes were first gated based on FSC/SSC characteristics and viability, followed by selection for HLADR and CD11b, which was then classified into CM, NCM, and IntM subsets using a CD14 *vs.* CD16 dotplot. Fluorescence Minus One (FMO) controls were used to guide gating.

**Supplementary Figure 2. Transcriptomic profiling and purity assessment of classical CD14⁺ monocytes. (A)** Schematic summary of the number of differentially expressed genes (DEGs) identified across the four cohorts (HD, gray; R, blue; NR_DTH⁻, red; NR_DTH⁺, orange). **(B)**  Representative dot plots showing CD14⁺CD3⁻gating used to evaluate the purity of magnetically isolated CD14⁺ monocytes by flow cytometry. **(C)** Quantification of CD14⁺CD3⁻ cell percentages across all samples (flow cytometry), paralleled by the relative abundance of immune cell populations inferred by CIBERSORTx-based deconvolution of RNA sequencing data, confirms predominant enrichment within the myeloid compartment.

**Supplementary Figure 3. Phenotypic characterization of DCs within the DC Vax product. (A)** Flow cytometry gating strategy used to identify myeloid DC subsets within the DCVax product, based on cell morphology and live cell marker. (**B)** Surface expression of co-stimulatory (CD80, CD83, CD86), antigen presentation (HLA-DR), and chemokine receptor (CCR7) markers in live-gated DCs, as assessed by flow cytometry. **(C)** Frequency of surface expression of inhibitory checkpoint molecules (PD-L1, PD-L2, TIM-3, VISTA, B7-H3) in live-gated DCs.

**Supplementary Figure 4. Characterization of BCR Clonotype Diversity in Responder and Non-Responder Patients. (A, B)** Scatter dot plots (mean ± s.e.m) showing diversity, mean clonotype frequency and number of clonotypes in the Q1 quantile of IgL𝜅 and IgL𝝺 CDR3 sequences from BCR repertoires of Rs and NRs. **(C, D)** Donut chart representations of IgH-CDR3 clonotype distribution for R and NR samples, respectively. The top 10 clonotypes are individually displayed. Total clonotype frequencies for each quantile (Q1–Q5), singleton, and doubleton categories are indicated in the legend. *P* value calculated using a two-tailed unpaired *t*-test.

**Supplementary Figure 5. Distribution of CD4⁺ T-cell subpopulations in Responder and Non-Responder Patients.**
**(A)** Stacked bar plots showing the relative frequency of CD4⁺ T-cell subpopulations in DCVax products from responders (R) and non-responders (NR), as determined by single-cell RNA sequencing. **(B)** Stacked bar plots displaying the distribution of CD4⁺ T-cell subsets across individual patients (P1–P17; blue, Responders; red, Non-Responders). CD4⁺ T-cell subpopulations were annotated using a marker-based single-cell classification framework following established reference atlases , and include naïve T cells (Tn), central memory T cells (Tcm), effector memory T cells (Tem), tissue-resident memory T cells (Trm), regulatory T cells (Treg), T follicular helper cells (Tfh), T helper 1 (Th1) cells, T helper 17 (Th17) cells, interferon-response T cells (Tisg), cytotoxic CD4⁺ T cells (Tc), proliferating T cells, activated T cells, and stress-response T cells (Tstr). No significant differences were observed between R and NR groups.
